# Multi‐Omics‐Based Autophagy‐Related Untypical Subtypes in Patients with Cerebral Amyloid Pathology

**DOI:** 10.1002/advs.202201212

**Published:** 2022-06-13

**Authors:** Jong‐Chan Park, Natalia Barahona‐Torres, So‐Yeong Jang, Kin Y. Mok, Haeng Jun Kim, Sun‐Ho Han, Kwang‐Hyun Cho, Xiaopu Zhou, Amy K. Y. Fu, Nancy Y. Ip, Jieun Seo, Murim Choi, Hyobin Jeong, Daehee Hwang, Dong Young Lee, Min Soo Byun, Dahyun Yi, Jong Won Han, Inhee Mook‐Jung, John Hardy

**Affiliations:** ^1^ Department of Neurodegenerative Disease UCL Queen Square Institute of Neurology University College London London WC1N 3BG UK; ^2^ Department of Biochemistry and Biomedical Sciences College of Medicine Seoul National University Seoul 03080 Republic of Korea; ^3^ Neuroscience Research Institute Medical Research Center College of Medicine Seoul National University Seoul 03080 Republic of Korea; ^4^ SNU Korea Dementia Research Center College of Medicine Seoul National University Seoul 03080 Republic of Korea; ^5^ Department of Bio and Brain Engineering Korea Advanced Institute of Science and Technology Daejeon 34141 Republic of Korea; ^6^ Division of Life Science State Key Laboratory of Molecular Neuroscience Molecular Neuroscience Center The Hong Kong University of Science and Technology Clear Water Bay, Kowloon Hong Kong 999077 China; ^7^ Hong Kong Center for Neurodegenerative Diseases Hong Kong Science Park Hong Kong 999077 China; ^8^ Guangdong Provincial Key Laboratory of Brain Science Disease and Drug Development HKUST Shenzhen Research Institute Shenzhen‐Hong Kong Institute of Brain Science Shenzhen Guangdong 518057 China; ^9^ Department of Laboratory Medicine Severance Hospital Yonsei University College of Medicine Seoul 03722 Republic of Korea; ^10^ European Molecular Biology Laboratory Genome Biology Unit Heidelberg 69117 Germany; ^11^ Department of Biological Sciences Seoul National University Seoul 08826 Republic of Korea; ^12^ Institute of Human Behavioral Medicine Medical Research Center Seoul National University Seoul 03080 Republic of Korea; ^13^ Department of Psychiatry College of medicine Seoul National University Seoul 03080 Republic of Korea; ^14^ Department of Neuropsychiatry Seoul National University Hospital Seoul 03080 Republic of Korea; ^15^ Department of Psychiatry Pusan National University Yangsan Hospital Yangsan 50612 Republic of Korea; ^16^ Biomedical Research Institute Seoul National University Hospital Seoul 03082 Republic of Korea

**Keywords:** Alzheimer's disease, autophagy, multi‐omics, peripheral blood, subtype, systems biology

## Abstract

Recent multi‐omics analyses paved the way for a comprehensive understanding of pathological processes. However, only few studies have explored Alzheimer’s disease (AD) despite the possibility of biological subtypes within these patients. For this study, unsupervised classification of four datasets (genetics, miRNA transcriptomics, proteomics, and blood‐based biomarkers) using Multi‐Omics Factor Analysis+ (MOFA+), along with systems‐biological approaches following various downstream analyses are performed. New subgroups within 170 patients with cerebral amyloid pathology (Aβ+) are revealed and the features of them are identified based on the top‐rated targets constructing multi‐omics factors of both whole (M‐TPAD) and immune‐focused models (M‐IPAD). The authors explored the characteristics of subtypes and possible key‐drivers for AD pathogenesis. Further in‐depth studies showed that these subtypes are associated with longitudinal brain changes and autophagy pathways are main contributors. The significance of autophagy or clustering tendency is validated in peripheral blood mononuclear cells (PBMCs; n = 120 including 30 Aβ‐ and 90 Aβ+), induced pluripotent stem cell‐derived human brain organoids/microglia (n = 12 including 5 Aβ‐, 5 Aβ+, and CRISPR‐Cas9 apolipoprotein isogenic lines), and human brain transcriptome (n = 78). Collectively, this study provides a strategy for precision medicine therapy and drug development for AD using integrative multi‐omics analysis and network modelling.

## Graphical abstract

Computational unsupervised classification using Multi‐Omics Factor Analysis (MOFA+) uncovers autophagy pathway‐related new subtypes within patients with cerebral amyloid pathology, with further biological validations using human induced pluripotent stem cell‐derived brain organoids or microglia, peripheral blood mononuclear cells, and human post‐mortem brains. Taken together, this manuscript herein suggests a multi‐omics‐based analytic platform of precision medicine approaches for Alzheimer's disease.

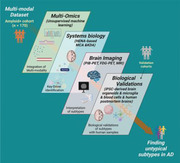

## 1 Introduction

Alzheimer's disease (AD) is one of the most common forms of neurodegenerative disorder characterized by progressive accumulation of cerebral beta‐amyloid (A*β*) plaques.^[^ ^1^ ^]^ Although the accumulation of A*β* plays a role in accelerating the progression of AD,^[^ ^2^ ^]^ AD is a multi‐factorial disorder for which genetic risk, immunity, and lipid metabolism are important contributors.^[^ ^3^ ^]^ In particular, the immune system is now considered one of the major factors in AD,^[^ ^3e^ ^]^ as AD pathology is accompanied by chronic inflammation with changes in innate immune cell populations in the brain, such as microglia, astrocytes, myeloid cells, dendritic cells, and other lymphocytic cells.^[^ ^4^ ^]^ Furthermore, a large number of known AD risk genes are related to immune responses including the complementary system or microglial function, which play a central role in amyloid pathways and neuronal death.^[^ ^3^, ^5^ ^]^ Several reports also demonstrated that peripheral inflammation contributes to pathological changes in AD due to the bidirectional transport of peripheral immune cells or cytokines and permeability of the blood‐brain‐barrier.^[^ ^4^, ^6^ ^]^ However, finding optimal drug‐targets is rather difficult given the complexity of AD progression caused by the diverse risk factors, despite the apparent impact of immunological factors. Several studies have suggested that the heterogeneity of AD exists, but very few have explored AD subtypes within affected patients.^[^ ^7^ ^]^ Hence, some integrated analytic approach overseeing diverse pathways is required to understand the intricate mechanisms of AD and unravel clues to the heterogeneity of its pathogenesis. However, a few studies have performed omics‐based subtyping trials; for example, Neff et al.^[^ ^8^ ^]^ only used RNA sequencing data from two different public cohorts (Religious Orders Study/Memory and Aging Project (ROSMAP) and Mount Sinai Brain Bank (MSBB)).

Recent technological advances have led to the new era in data analyses with large volumes of data from different multi‐omics layers.^[^ ^9^ ^]^ For instance, unsupervised integration of multi‐omics datasets has identified subtypes of many heterogeneous cancers, such as pancreatic ductal adenocarcinoma, renal cell carcinoma, and stomach adenocarcinoma.^[^ ^9^, ^10^ ^]^ However, methods for the integration of heterogeneous datasets or various data‐types are still lacking. Interestingly, the latest method called multi‐omics factor analysis+ (MOFA+), a computational method for unsupervised integration decomposing the sources of data heterogeneity, was developed by a group in the European Molecular Biology Laboratory (Heidelberg, Germany) to address this problem.^[^ ^11^ ^]^ They demonstrated that MOFA+ is able to identify major sources of variation in heterogeneous disease and reveal novel putative molecular drivers of each possible subtype. Furthermore, it enables the reconstruction of multi‐omics factors (MOFs) and merges different patterns of missing data. Since it is hard to collect all information without missing values and match all data‐types for multi‐omics layers, MOFA+ is a great tool for a wide range of multi‐omics studies.

In this study, MOFA+ was applied to our multiple datasets (targeted‐sequencing, miRNA transcriptome, proteomics, and other blood‐based biomarkers) from blood samples of patients with cerebral amyloid pathology. Through this unsupervised data integration, we aimed to find unknown subtypes of AD within patients. Moreover, using a systems‐biological approach, we explored the characteristics of subtypes and possible key‐drivers. Peripheral blood mononuclear cells (PBMCs) and induced pluripotent stem cell (iPSC)‐derived human brain organoids were used for further validation. Taken together, we herein suggest a multi‐omics‐based analytic platform for AD precision medicine approaches.

## 2 Results

### 2.1 Demographics of the Participants

The demographic data of participants with cerebral amyloid pathology (Pittsburgh compound B‐positron emission tomography scan positive cases; PiB+) or validation cohorts are described in Tables S1–S4, Supporting Information. For the multi‐omics study, the population comprised cognitively normal participants (CN+, *n =* 40), and patients with mild cognitive impairment (MCI+, *n =* 65) and AD dementia (DEM+, *n =* 65). Since the aim was to discover new subtypes within patients with A*β* in the brain, PiB negative cases (PiB−) were not included in the multi‐omics model. For the statistical validation of MOFA+ tool, a Chinese cohort (*n =* 106 patients with AD; *n =* 74 CN) was used (see Table S2, Supporting Information). For the validation of key‐drivers from multi‐omics analysis, two additional cohorts, including PiB− cases (cohort for peripheral validation: *n =* 30 PiB−, *n =* 90 PiB+, total *n =* 120; cohort for central validation: *n =* 5 PiB−, *n =* 5 PiB+, total *n =* 10), and public NCBI GEO database (total *n =* 78; *n =* 32 CN, *n =* 46 AD; GSE109887) were used (see Tables S3–S4, Supporting Information).

### 2.2 The Overall Design of the Study

**Figure 1:**
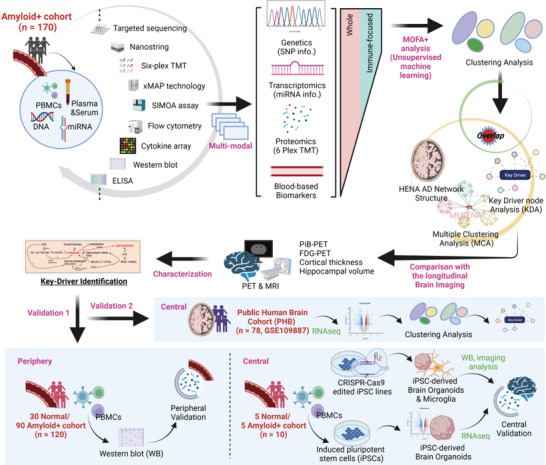
Graphical summary of the study concept. A wide variety of experimental and analytic tools were utilized prior to the machine learning processes, using human blood‐derived biopsied specimen (PBMC, DNA, plasma, serum, miRNA) from 170 patients with cerebral amyloid pathology. The unsupervised machine learning (MOFA+) and clustering analyses were performed followed by four multimodal generations. After clustering analyses, each of the clusters (untypical subtypes within the patients) was characterized using the systems biological approaches. Potential key‐drivers contributing to the clusters (the overlapped drivers with the MCA & KDA based on HENA AD network structure) were selected and analyzed using the longitudinal brain‐imaging datasets including PiB‐PET, FDG‐PET, and MRI scans. After characterization of the key‐drivers, final targets were further validated using human PBMC samples, iPSC‐derived brain organoids, CRISPR‐Cas9 base edited iPSC‐derived organoid or microglia, and human post‐mortem brains (validations 1 and 2).

A graphical concept of this study is presented in **Figure** **1** and the overall step‐by‐step approaches for multi‐omics analysis are shown in Figure S1, Supporting Information. For the multi‐omics analysis, various experiments and analyses were performed using human blood‐derived biopsied specimens (plasma, serum, miRNA, DNA, and PBMCs) (Figure 1; upper section). Four datasets (genetics, targeted sequencing data; miRNA transcriptomics, Nanostring nCounter miRNA analysis; proteomics, six‐plex TMT‐based blood proteomics; blood‐based biomarkers, immunoassays, or colorimetric assays) were generated and applied as multi‐omics layers (see Figure S1a, Supporting Information). Two different models were generated by MOFA+ to identify data subtypes. First, a multi‐omics model using whole datasets (multi‐omics‐based total profiling model for AD; M‐TPAD) was created to represent every possible mechanism of AD. This M‐TPAD model included 1133 single nucleotide variants (SNVs) within 125 genes, 109 miRNAs, 398 proteins, and 136 blood‐based biomarkers. Second, we generated a multi‐omics based‐immune profiling model for AD (M‐IPAD) that focused on immune‐related targets sorted by the public Immport DB database.^[^ ^12^ ^]^ For the M‐IPAD model, 658 SNVs within 76 genes, 109 miRNAs, 183 proteins, and 123 blood‐based biomarkers were used. Further details of the demographic data used in both models are shown in Tables S5 and S6, Supporting Information.

For both M‐TPAD and M‐IPAD models, quality‐controls and normalization for each dataset were performed to insert them into the learning protocol by MOFA+ in R software (see Figure S2, Supporting Information). The generated MOFs (the factors capturing the global sources of variability in the data and ordinating cells along a 1D axis centered at zero; the relative positions of samples are converted as numeric values for the downstream analysis)^[^ ^11a^ ^]^ and automatically imputed datasets were used for clustering and further downstream analyses (see Figure S1b, Supporting Information, upper two graphs). Characterization of each subtype was conducted by systems‐biological approaches (see Figure S1b, Supporting Information, lower two graphs). Top‐weighted targets and their association were evaluated by our biological network model through systems‐biological approaches. For validation, the significance of key‐drivers from the multi‐omics analysis was validated both in PBMCs (*n =* 120 including 30 PiB− and 90 PiB+) and iPSC‐derived human brain organoids (*n =* 10 including 5 PiB− and 5 PiB+). In addition, CRISPR‐Cas9‐based gene‐edited iPSC lines (Apolipoprotein E (ApoE) ɛ3/ɛ3 iPSC, parental line; ApoE ɛ4/ɛ4 iPSC, isogenic line) were also used for the generation of iPSC‐derived microglia and 3D brain assembloids (brain organoids with iPSC‐derived microglia). Finally, the clustering tendency was further validated using a different public NCBI GEO cohort (GSE109887), including human post‐mortem brain transcriptome data (*n =* 78 including 46 AD and 32 CN) (Figure 1).

### 2.3 Application of MOFA+ and Subtyping of Patients

**Figure 2:**
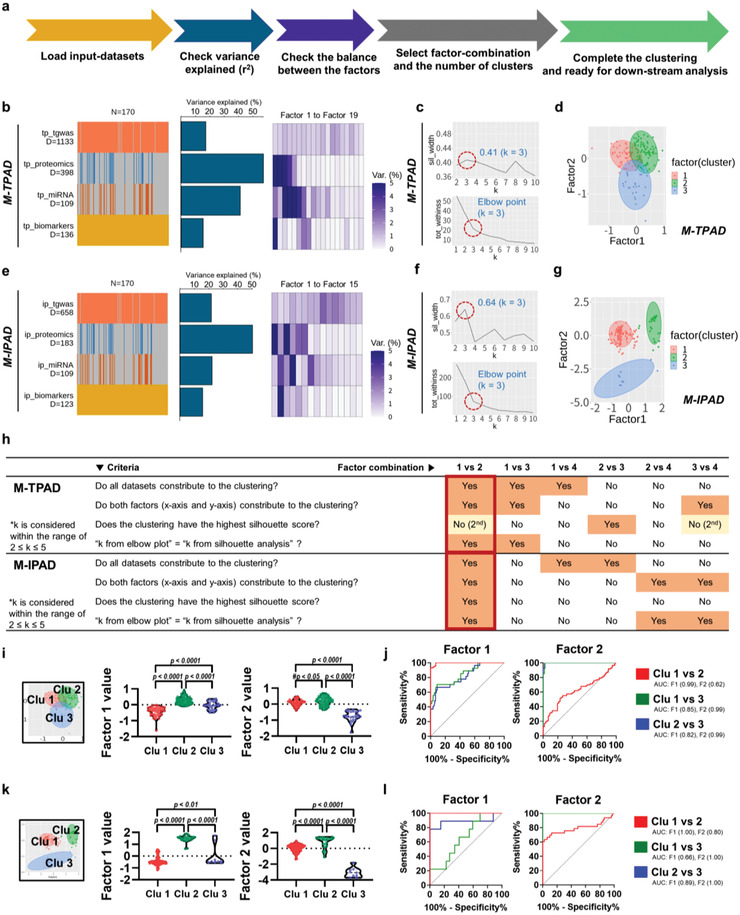
Application of MOFA+ to a study of AD (M‐TPAD and M‐IPAD model). a) Process for finding new AD subtypes using the MOFA+ and ggplot2 in R software. The methods for selection of factor‐combination and determination of the number of clusters are described in detail in Figure 3. b,c) Overview of the M‐TPAD and M‐IPAD models. The types of dataset layers (left), cumulative proportion of total variance explained (middle), and proportion of variance explained of individual factors (right) are shown. d) Silhouette and elbow plot analysis to determine the number of clusters and appropriate factor‐combination for the M‐TPAD model. e) Generated clusters (k = 3) by k‐medoids clustering for the M‐TPAD model. f) Silhouette and elbow plot analysis to determine the number of clusters and appropriate factor‐combination for the M‐IPAD model. g) Generated clusters (k = 3) by k‐medoids clustering for the M‐IPAD model. h) Application of standardized criteria for determination of the factor‐combination and number of clusters (k value). Both M‐TPAD and M‐IPAD models showed the best combination between Factor 1 and Factor 2 with the same k value (k = 3). i) Comparison of MOFs between the clusters from the M‐TPAD model. P‐values were obtained from ANOVA with Tukey's post‐hoc test. ^#^P‐values were from unpaired *t*‐test. j) ROC curve analysis using the values of factor 1 or factor 2 to discriminate between the clusters from the M‐TPAD model. See details in Table S7, Supporting Information. k) Comparison of MOFs between the clusters from the M‐IPAD model. P‐values were obtained from ANOVA with Tukey's post‐hoc test. l) ROC curve analysis using factor 1 or factor 2 to discriminate between the clusters from the M‐IPAD model. See details in Table S7, Supporting Information. Abbreviations: ip, M‐IPAD; tgwas, targeted sequencing data from genome‐wide associated loci study; tot_withinss, total within‐cluster sum of squares; tp, M‐TPAD; sil_width, silhouette width; Var, variance explained; AUC, area under curve; Clu, cluster; F1, factor 1; F2, factor 2.

After quality‐controls and normalization of the datasets (see Figures S1 and S2, Supporting Information), MOFA+ was used as follows: i) loading the four datasets, ii) checking the variance explaining each dataset, iii) comparing the balances between the MOFs, iv) selecting appropriate combination of two MOFs, v) estimating the optimal number of clusters and finding appropriate subtypes for PiB+ (**Figure** **2**a–g). After all datasets were successfully loaded (Figure 2b,e, left), average percentage of explained variances was confirmed (> 20%) (Figure 2b,e, middle). Moreover, the variances were laid out by each MOFs, respectively (Figure 2b,e, right). For the steps (iii) to (v) (Figure 2b,e right and Figure 2c,d,f,g), standardized criteria were used for determining clusters within the patients with PiB+ (Figure 2h). First, elbow plots and silhouette curves of each factor‐combination were visualized for estimating the optimal number of clusters (Figure 2c,f, see Figure S3, Supporting Information). Furthermore, the k value was determined within the range of 2–5. After clustering with the k‐medoids method (see Figure S3, Supporting Information), each factor‐combination underwent assessments by the standardized criteria to select the best clusters (Figure 2h). Through the four standards (Figure 2h), the combination between factor 1 and factor 2 was selected as the best option for both M‐TPAD and M‐IPAD models (Figure 2h; see Figure S3, Supporting Information). All scores of clusters were well‐balanced within the range of ±10% from total average silhouette width (0.40–0.43 for M‐TPAD, 0.58–0.66 for M‐IPAD) (see Figure S3c,d, Supporting Information). Especially, clustering of the M‐IPAD model showed a fairly high average silhouette score (> 0.5) (see Figure S3d, Supporting Information).

### 2.4 Preliminary Evaluation of M‐TPAD and M‐IPAD Models

Demographic data of the M‐TPAD and M‐IPAD clusters are shown in Tables S5 and S6, Supporting Information. First, the MOFs (factors 1 and 2) of the M‐TPAD model were significantly different between all the clusters (Figure 2i). More, receiver operating characteristic (ROC) curve analysis showed that all comparisons of clusters had significant *p*‐values (*p* < 0.05) and high area under the curves (AUC) (Figure 2j, see Table S7, Supporting Information). Similar to M‐TPAD, MOFs (factors 1 and 2) of the M‐IPAD model were dramatically different between all the clusters (Figure 2k) and ROC curves showed high differences between all comparisons (Figure 2l, see Table S7, Supporting Information). These results suggest that our clusters are well‐divided and seemed to be ready for the downstream analysis. However, we needed to confirm that the generated clusters did not simply rely on the well‐known AD‐related factors. Interestingly, almost all these factors (age, sex, apolipoprotein E status, brain A*β*, mini‐mental state exam scores, hippocampal volume (Hva), cortical thickness, brain glucose metabolism) in the clusters from M‐TPAD and M‐IPAD did not show any differences (see Figure S4, Supporting Information), and were not correlated with the MOFs (see Figure S5, Supporting Information), except for cluster 3 from the M‐IPAD model, which showed significantly higher accumulation of brain A*β* than clusters 1 and 2 (see Figure S4c, Supporting Information, first graph). Thus, we speculated that our clusters were generated by hidden, but important, factors constructing our multi‐omics model.

### 2.5 Key Targets Contributing to the Clustering of the M‐TPAD and M‐IPAD Models

**Figure 3:**
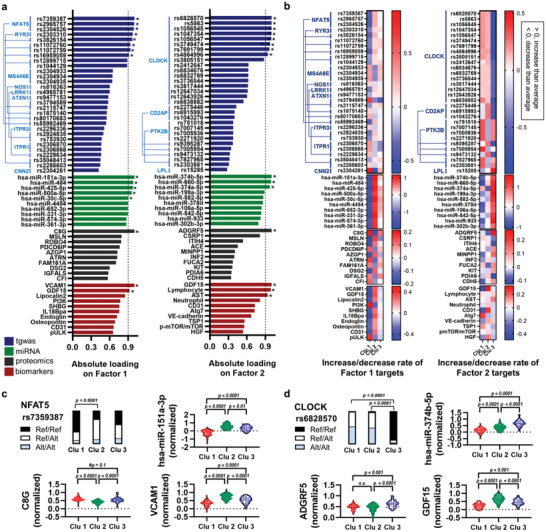
Key‐targets contributing to the clustering of the M‐TPAD model. a) List of top‐rated targets (top 30 for SNVs and top 10 for others) on factor 1 or factor 2. Absolute loading values were used for the list. Asterisks denote superlative targets (absolute loading value > 0.9) of each dataset. b) Heatmaps for the top‐rated targets showing increased (> 0) or decreased (< 0) rate compared with the average value of the whole cohort. For the tgwas dataset, the rate means increased (> 0) or decreased (< 0) incidence of each SNV compared with the average incidence in the whole cohort. c,d) Comparison of the highest weighted targets (factor 1 or factor 2) between the clusters from the M‐TPAD model. P‐values were obtained from ANOVA with Tukey's post‐hoc test or Chi‐square test. ^#^ *P*‐values were from unpaired *t*‐test. Abbreviations: Alt, the allele in the alternative genome; Clu, cluster; Ref, the allele in the reference genome; tgwas, targeted sequencing data of genome‐wide associated loci study.

**Figure 4:**
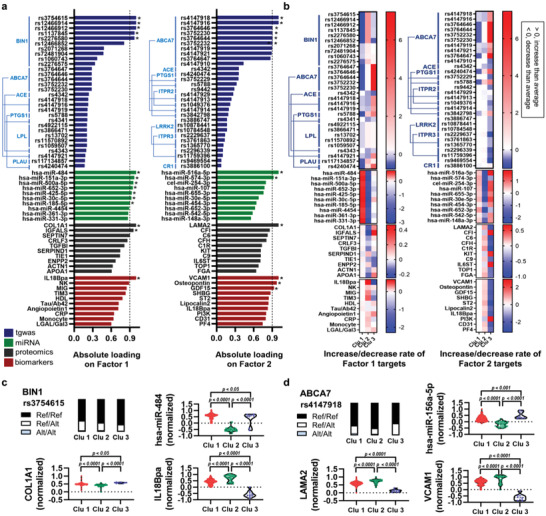
Key targets contributing to the clustering of the M‐IPAD model. a) List of top‐rated targets (top 30 for SNVs and top 10 for others) on factor 1 or factor 2. Absolute loading values were used for the list. * denotes superlative targets (absolute loading value > 0.9) of each dataset. b) Heatmaps for the top‐rated targets showing increased (> 0) or decreased (< 0) rate compared with the average value of the whole cohort. For the tgwas dataset, the rate means increased (> 0) or decreased (< 0) incidence of each SNV compared with the average incidence of the whole cohort. c,d) Comparison of the highest weighted targets (factor 1 or factor 2) between the clusters from the M‐IPAD model. P‐values were obtained from ANOVA with Tukey's post‐hoc test or Chi‐square test. ^#^P‐values were from unpaired *t*‐test. Abbreviations: Alt, the allele in the alternative genome; Clu, cluster; Ref, the allele in the reference genome; tgwas, targeted sequencing data of genome‐wide associated loci study.

We listed the top‐rated targets contributing to the clustering of the M‐TPAD and M‐IPAD models (**Figures** **3** and **4**). Top 30 (for SNVs) or top 10 key targets (for other datasets) from the M‐TPAD model are listed and their absolute values loaded on each MOF are presented in Figure 3a. For comparison between the clusters, heatmaps representing increased (> 0) or decreased (< 0) rate for the incidences of each SNV or for the levels of top‐rated targets compared with the average value of the whole cohort are shown in Figure 3b. In addition, comparisons of the highest weighted targets are shown in Figure 3c,d.

Similar to the M‐TPAD model, all of the same analyses for M‐IPAD model were conducted (**Figure** **4**a,b). As expected, almost all of the highest weighted targets from the M‐IPAD model were significantly different between the clusters (Figure 4c,d). Moreover, all of the highest weighted targets from both models were dramatically correlated with MOFs (see Figure S6a,b, Supporting Information), and ROC curve analysis using all of the highest weighted targets showed significant *p*‐values and high AUCs (see Figure S6c and Table S8, Supporting Information). However, since MOFA+ is a computational unsupervised integration that cannot include a biological interpretation, further systems‐biological approaches for M‐TPAD and M‐IPAD models were still needed.

### 2.6 Characterization of Subtypes within AD Patients by Systems‐Biological Approaches

First, we performed STRING analysis (canonical protein‐protein interaction (PPI) analysis) and Integrated Interactions Database analysis (context‐specific PPI analysis),^[^ ^13^ ^]^ analytic tools for functional protein‐protein association networks, to identify molecular networks of the top‐rated targets from MOF (factor 1 or 2) both in the M‐TPAD and M‐IPAD models (see Figures S7 and S8, Supporting Information). Almost all of the top‐rated targets showed significant interactions in each MOF (M‐TPAD or M‐IPAD factor 1, 2); particularly, the targets consisting of factor 2 of M‐IPAD showed the highest functional association (number of nodes, number of edges, and average node degree) and the highest number of PPIs (see Figure S7d and Figure S8, Supporting Information). This suggests that almost all the top‐rated targets consisting of our multi‐omics models are clearly linked with each other.

**Figure 5:**
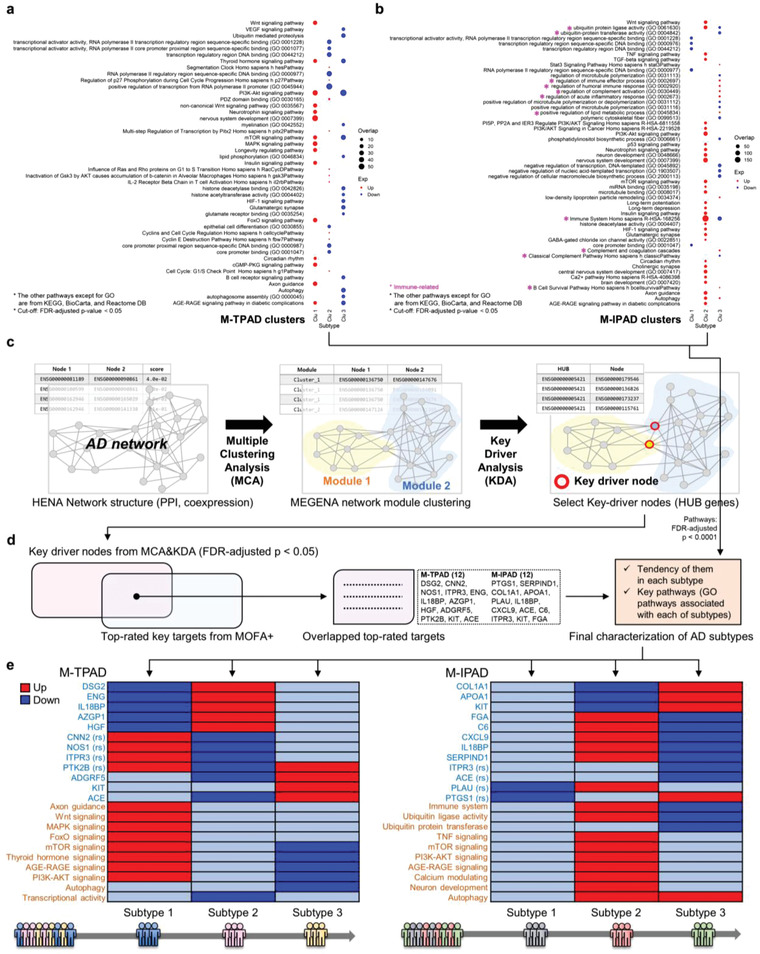
Final characterization of subtypes within AD patients by convergence between multi‐omics analysis and systems‐biological approaches. a,b) Enriched pathway analysis using the whole dataset to reveal key pathways of each cluster in the M‐TPAD and M‐IPAD models. The normalized activities (threshold > 0.5 or < −0.5) of each target were used as input. The pathways were selected from GO, KEGG, BioCarta, and Reactome databases. P‐values, FDR‐adjusted *p* < 0.05. c) Identification of key driver nodes by KDA following MCA. d) Final steps for characterization of subtypes within AD patients. The overlapped top‐rated targets with the key driver nodes from KDA were finally selected as key molecules. Moreover, key pathways were singled out again by the cut‐off with FDR‐adjusted *p*‐value < 0.0001. Exceptionally, the term “Immune system” from Reactome database (R‐HSA‐168256) was included in the final phenotypes because it had the highest enriched pathway score although the FDR‐adjusted *p*‐value was between 0.0001 and 0.05. e) Final characterization of the subtypes within AD patients by convergence between multi‐omics analysis and systems‐biological approaches. Abbreviations: Clu, cluster; DB, database; GO, gene ontology; PPI, protein‐protein interaction.

To investigate the biological role of each cluster, we performed enriched pathway analysis using our targets that showed a significant increase or decrease rate (threshold > 0.5 or < −0.5, respectively) compared with the average value of the whole cohort to reveal important pathways of each cluster in both M‐TPAD and M‐IPAD (**Figure** **5**a,5b). Using publicly available databases (gene ontology (GO), Kyoto Encyclopedia of Genes and Genomes (KEGG), BioCarta, and Reactome), significantly enriched pathways were selected. Interestingly, many immune‐related pathways (immune systems, complement pathways, lipid metabolic process, complement activation, among others) were identified in the M‐IPAD model (Figure 5b). Furthermore, clusters 1 and 3 of the M‐TPAD model showed dramatically opposite characteristics, despite they both comprised PiB+ participants without any differences in their cognitive functions, age, sex, ApoE type, or brain status (Figure 5a, see Figure S4 and S5, Supporting Information).

In addition, cluster 2 of the M‐IPAD model showed distinctive characteristics compared with clusters 1 and 3 (Figure 5b). Next, we performed MCA followed by key driver node analysis (KDA) to identify the key‐drivers among our top‐rated targets (Figure 5c). The HENA network model and MEGENA approach were used to identify key driver nodes within the network model.^[^ ^14^ ^]^ The overlapped targets between the driver nodes and our top‐rated targets from the multi‐omics models were finally selected as drivers in our models (Figure 5d). With the main pathways from the enriched pathway analysis (false discovery rate (FDR)‐adjusted *p*‐value < 0.0001), the key drivers were finally chosen as the main phenotypes of our multi‐omics models (Figure 5e). Thus, final characterization of the subtypes within the AD patients was completed by the convergence between multi‐omics analysis and systems‐biological approaches.

### 2.7 Association between Longitudinal Changes in the Brain and Key‐Drivers of AD Subtypes

**Figure 6:**
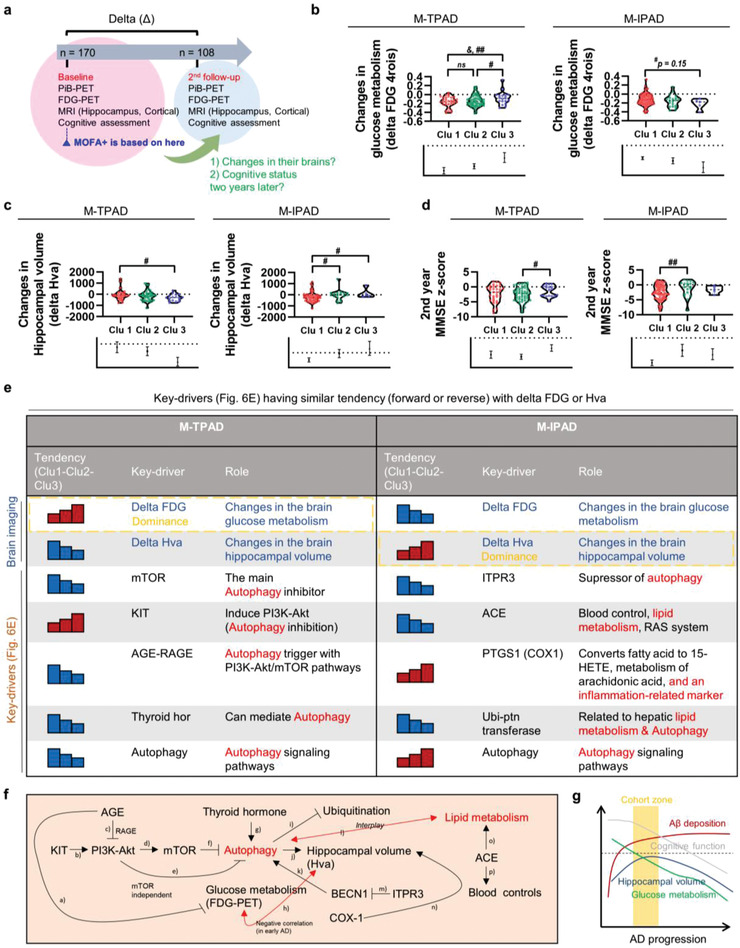
Interpretation of AD subtypes along with longitudinal changes in the brain. a) Timeline of the longitudinal study (baseline and 2nd year follow‐up) of 108 participants. Delta represents differences between the second and first measurement values. b) Comparison of longitudinal changes of glucose metabolism (FDG‐PET) between the subtypes. ^#^ *p* < 0.1, ^##^ *p* < 0.05, two‐sided independent *t*‐test (for M‐TPAD) or Mann‐Whitney test (for M‐IPAD); ^&^ *p* < 0.1, ^&&^ *p* < 0.05, ANOVA with Tukey's post‐hoc test. c) Comparison of longitudinal changes of Hva between the subtypes. ^#^ *p* < 0.1, ^##^ *p* < 0.05, two‐sided independent *t*‐test (for M‐TPAD) or Mann‐Whitney test (for M‐IPAD); ^&^ *p* < 0.1, ^&&^ *p* < 0.05, ANOVA with Tukey's post‐hoc test. d) Comparison of the 2nd year MMSE z‐scores between the subtypes. ^#^ *p* < 0.1, ^##^ *p* < 0.05, two‐sided independent *t*‐test (for M‐TPAD) or Mann‐Whitney test (for M‐IPAD); ^&^ *p* < 0.1, ^&&^ *p* < 0.05, ANOVA with Tukey's post‐hoc test. e) Key‐drivers having similar (sKeys) patterns with delta FDG‐PET or Hva (forward or reverse tendency from cluster 1 to cluster 3). Arrows indicate increase or decrease tendency from cluster 1 to cluster 3. f) Associations between sKeys from both M‐TPAD and M‐IPAD models. Autophagy‐related pathways were mainly involved in the pathway map. g) Hypothetical AD progression plot. Conjectured participant distribution range are marked as yellow. Abbreviations: Clu, cluster; MMSE z‐score, mini‐mental state examination with the correction for age, sex, and education level.

As our clustering had no noticeable relationship with the well‐known AD‐related features (cross‐sectionally), we speculated that our clusters were generated by hidden factors that could be characterized by the combination of systems‐biological approaches. However, if significant longitudinal changes in the brain occur that also could contribute to AD clustering, it would not be possible to collect such molecular information. Therefore, 108 participants among the initial cohort (*n =* 170) were recruited again to undergo additional comprehensive evaluations and multi‐modal brain imaging, such as PiB‐PET, FDG‐PET, and MRI (**Figure** **6**a). There have been many changes in the condition of participants for 2 years (cognitive decline was observed in 63.5% of patients, PiB increase was observed in 77.9% of patients, FDG decrease was observed in 89.8% of patients, Hva decrease in 73.8% of patients, and clinical dementia rating changes in 26.8% of patients) (Figure S9, Supporting Information). Interestingly, comparisons of the changes in FDG‐PET or Hva MRI (delta values) data between AD subtypes revealed significant differences between the clusters both in the M‐TPAD and M‐IPAD models (Figure 6b–6d). Next, we further tried to find which key‐drivers were relevant to these pathological changes (decreasing or increasing trend from cluster 1 to cluster 3) in the brains (Figure 6e), which were named “key‐drivers having similar patterns with delta FDG‐PET or Hva” (sKeys). Surprisingly, most sKeys were mainly involved in autophagy pathways (Figure 6f). Furthermore, the activation/inhibition tendencies (lines of Figure 6f) according to the references (Table S9, Supporting Information) corresponded to our decreasing or increasing trends of sKeys from cluster 1 to cluster 3.^[^ ^15^ ^]^

Noteworthy, FDG‐PET (from cluster 1 to cluster 3; increasing FDG in M‐TPAD, decreasing FDG in M‐IPAD) and Hva (from cluster 1 to cluster 3; decreasing Hva in M‐TPAD, increasing Hva in M‐IPAD) tended to show the opposite trend in M‐TPAD and M‐IPAD, which could be explained by the fact that our KBASE cohort is mostly distributed in early AD stage (Figure 6g, yellow‐shaded) according to the hypothetical AD progression plot previously published.^[^ ^15h^ ^]^ Since there have been several reports of autophagy‐related pathways being highly associated with Hva changes (especially through BECN1, COX1), glucose metabolism (through AGE‐RAGE pathways), and neurodegeneration, our interpretation of the data provide reasonable evidence for the appropriate MOFA+ clustering.^[^ ^15a,15b,15h,15k,15n^ ^]^ Thus, beyond the multi‐omics analysis and systems biological approaches, we further concluded that the subtypes of AD from M‐TPAD and M‐IPAD models are even associated with longitudinal changes in the brains of AD patients, and that autophagy‐related pathways are the main contributors for the clustering.

### 2.8 Validation of Impact of Autophagy on AD

**Figure 7:**
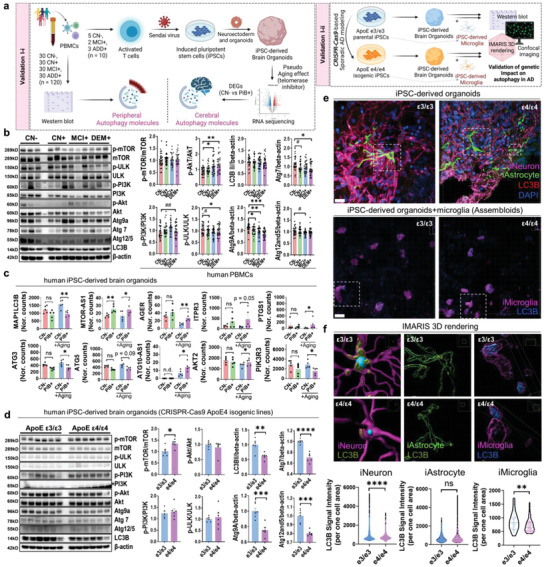
The impact of autophagy on AD. a) A graphical summary of experimental settings for the validation cohort (*n =* 120 for PBMC, *n =* 10 for iPSC‐derived brain organoids, CRISPR‐Cas9‐ based ApoE isogenic lines). For pseudo aging effect (mimicking longitudinal analysis similar to Figure 6), iPSC‐derived brain organoids were treated with a telomerase inhibitor (MST‐312). b) Autophagy‐related molecules were significantly altered in PBMCs from the patients with cerebral amyloid pathology. A total of 120 samples from 10 cohorts (each cohort comprised 12 samples) were quantified for the validation of autophagy‐related molecules in PBMC samples. *p < 0.05, **p < 0.01, and ***p < 0.001 by one way ANOVA with Tukey post‐hoc test. ^#^ *p* < 0.05 and ^##^ *p* < 0.01 by unpaired *t*‐test (*n =* 30, CN without brain amyloid pathology, CN−; *n =* 30 CN with brain amyloid pathology, CN+; *n =* 30 MCI with brain amyloid pathology, MCI+; *n =* 30 dementia patients with brain amyloid pathology, DEM+). c) Pseudo‐aging effect (MST‐312) induces changes in the expression of autophagy‐related gene. Ten iPSC‐derived brain organoids were used for the RNA sequencing analysis (*n =* 5, CN−; *n =* 2, MCI+; n = 3, DEM+; total *n =* 10). *p < 0.05, **p < 0.01, calculated using limma in R. d) Autophagy‐related molecules were significantly altered in the ApoE ɛ4/ɛ4 isogenic brain organoids compared to ɛ3/ɛ3 parental brain organoids (*p < 0.05, **p < 0.01, ***p < 0.001, and ****p < 0.0001 by independent *t*‐test). e) High‐resolution images by confocal microscopy using iPSC‐derived brain organoids and brain assembloid (organoid + microglia). For upper images, GFAP (astrocyte, green); LC3B (autophagy marker, red), MAP2 (neuron, pink), and DAPI (nucleus, blue) were used. For lower images, TREM2 (microglia, pink) and LC3B (autophagy marker, blue) were used. Scale bar = 20µm. White boxes indicate 3D rendering position for Figure 7f. *n =* 4 organoid or brain assembloid tissues were used for imaging. f) IMARIS 3D rendering and LC3B quantification. In sum, *n =* 3250 ɛ3/ɛ3 neurons, *n =* 2391 ɛ4/ɛ4 neurons, *n =* 538 ɛ3/ɛ3 astrocytes, *n =* 148 ɛ4/ɛ4 astrocytes, *n =* 32 ɛ3/ɛ3 microglia, *n =* 76 ɛ4/ɛ4 microglia were quantified individually. **p < 0.01 and ****p < 0.0001 by independent *t*‐test.

Because autophagy‐related pathways were major key‐drivers in our multi‐omics models, we needed to confirm that our PiB+ patients from KBASE cohort were impacted by autophagy‐pathways both in the periphery and the brain compared to PiB− patients. Moreover, dysregulation of autophagy is one of the contributors to AD pathology; little is known about the links between peripheral autophagy and AD or the direct association of longitudinal brain changes with autophagy‐related molecules in the brain. For the validation of autophagic dysfunction in AD, certain additional cohorts with PiB− cases (cognitively normal without cerebral amyloid pathology; CN−) were included (PBMC samples, *n =* 120 including 30 PiB− and 90 PiB+; iPSC‐derived human brain organoids, *n =* 10 including 5 PiB− and 5 PiB+), and PiB− and PiB+ were comparatively analyzed (**Figure** **7**a, validation I‐i; related to Figure 7b,c). Moreover, we further tested the impact of most popular AD risk gene (ApoE genotypes) on autophagy pathways through our 3D iPSC‐derived brain organoids or iPSC‐derived assembloid system (mixed culture model; organoid + microglia) using CRISPR‐Cas9‐based sporadic AD modeling iPSC lines (ApoE ɛ3/ɛ3 iPSC, parental line; ApoE ɛ4/ɛ4 iPSC, isogenic line) (Figure 7a, validation I‐ii; related to Figure 7d–7f).

Interestingly, autophagy‐related molecules (Atg7, Atg12/5, p‐ULK/ULK, and Atg9) in the PBMCs were significantly down‐regulated with the progression of AD (Figure 7b). Moreover, PI3K/Akt signaling pathway (p‐PI3K/PI3K or p‐Akt/Akt) was enhanced compared to CN− whereas the levels of p‐mTOR/mTOR were not changed (Figure 7b). This phenomenon implies that PI3K/Akt regulates peripheral autophagy‐related molecules using the mTOR‐independent pathways; this is consistent with the inhibitory arrow “e” in Figure 6f. Therefore, the changes in peripheral autophagic pathways in the PBMCs may induce various phenotypes in the patients with cerebral amyloid pathology, compared to those in normal subjects without cerebral amyloid pathology. Subsequently, for the validation of central autophagic changes in AD, our previous RNA sequencing data with human iPSC‐derived brain organoids were re‐analyzed.^[^ ^3f^ ^]^

As the organoids were treated with the telomerase inhibitor MST‐312 for pseudo‐aging effect (aging process), we considered this analytical setting to correspond to our longitudinal analysis in Figure 6. Similar to the periphery, the expressions of autophagy‐related genes (ATG3, ATG5) and anti‐sense genes (ATG10‐AS1) were significantly altered in the PiB+ cohort with MST‐312 treatment, but not in the PiB+ cohort without MST‐312 treatment, compared to those in the CN− cohort (Figure 7c). However, in contrast with the peripheral autophagic system, MAP1LC3B (a coding gene for autophagy‐related protein LC3B) was also decreased in the PiB+ cohort with MST‐312 treatment, but not in the PiB+ cohort without MST‐312 treatment, compared to that in the CN− cohort (Figure 7c). Moreover, the anti‐sense MTOR gene (MTOR‐AS1) had less effect on the MST‐312‐treated organoids than on the non‐treated organoids (Figure 7c). This indicates that central autophagy systems are mediated by mTOR‐dependent autophagy pathways whereas peripheral systems are not. Furthermore, our other key‐drivers from Figure 6, such as AGER, ITPR3, and PTGS1, also exhibited significant differences between CN− and PiB+ in the MST‐312‐treated organoids compared to those in the non‐treated organoids (Figure 7c). In summary, Figure 7a–7c clearly shows that although we cannot argue that autophagy is the only factor determining clustering because our results were derived from unsupervised machine learning with a variety of datasets, the complex link between the progression of AD and peripheral and central autophagic regulatory systems may contribute to untypical subtypes among the patients with cerebral amyloid pathology, which could be a key‐driver for our multi‐omics models.

Next, we performed western blotting using ApoE ɛ3/ɛ3 and ɛ4/ɛ4 iPSC‐derived organoids (Figure 7d). In contrast with the results from PBMCs (Figure 7b and Figure S10a, Supporting Information), we observed that the most popular sporadic AD risk gene, ApoE, also can modulate mTOR‐dependent autophagic pathways and LC3B molecules, but cannot regulate PI3k‐Akt pathways (Figure 7d). Moreover, the confocal microscopy images and 3D rendering results by IMARIS software revealed that iPSC‐derived neurons and microglia from e4/e4 show a deficit of LC3B in our iPSC‐derived 3D brain assembloid system (Figure 7e,f, Figure S10b, Supporting Information). This phenomenon implies that AD clusters can also be affected by ApoE genotypes, but simultaneously let us know that is not the only one reason for our clustering results, because our clusters from M‐TPAD and M‐IPAD do not show differences in ApoE genotypes between the clusters (Tables S5 and S6, Supporting Information) and seem to be affected by PI3K‐Akt pathways (mTOR independent; see “e” pathway in Figure 6f and Figure 7b) rather than mTOR‐dependent autophagic pathways. Of course, it is difficult to argue that mTOR pathways or ApoE genotypes are not associated with our clusters, because i) PI3k‐Akt‐mTOR pathway was one of the enriched pathways in our clustering results and ii) lipid metabolic pathways are major key‐driver pathways from our M‐IPAD model (Figure 6e). In summary, we can conclude that the outcome of clustering in AD have been influenced by multiple autophagy‐related pathways both in the periphery and central nerve systems and speculate that mTOR and PI3k‐Akt pathways are differently regulated in accordance with their biological locations.

### 2.9 Validation of Clustering Results

**Figure 8:**
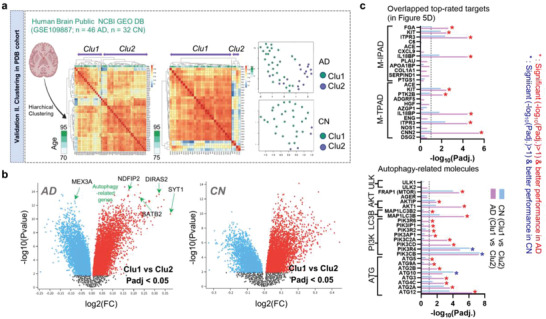
Clustering validation among the patients with AD. a–c) Clustering validation using a public transcriptome database with the post‐mortem brains (total *n =* 78; 46 AD and 32 CN). Volcano plot presents the significant DEGs between each cluster (AD cluster 1 vs cluster 2 or CN cluster 1 vs cluster 2) with the adjusted p‐values (Padj < 0.05). The arrow in the volcano plots indicates autophagy‐related genes. Bar graphs show the significance of the overlapped top‐rated targets from our multi‐omics models and autophagy‐related molecules (cut‐off, ‐log_10_(Padj) > 1).

Finally, we performed clustering and transcriptome analysis using a public human brain transcriptome dataset from the Gene Expression Omnibus (GEO) database (GSE109887) (**Figure** **8**a). As expected, we observed clear dichotomized clusters in the patients with AD, but not in the CN subjects (Figure 8a). Interestingly, the top‐ranked significant DEGs in AD (AD cluster 1 vs AD cluster 2), such as MEX3A (autophagic vesicle protein), NDFIP2 (an endosomal protein with a role in vesicular trafficking or ubiquitin ligase activator), DIRAS2 (autophagy‐mediated cell‐death by inhibition of Akt‐mTOR), SYT1 (synaptic vesicle trafficking), and SATB2 (hypoxia‐induced autophagy) were autophagy‐related genes but those in the CN subjects (CN cluster 1 vs CN cluster 2) were not (Figure 8b).^[^ ^16^ ^]^ Moreover, numerous top‐rated targets from our multi‐omics models (M‐TPAD, M‐IPAD) and autophagy‐related molecules were also significantly different between Cluster 1 versus Cluster 2 in AD compared to Cluster 1 versus Cluster 2 in CN (Figure 8c). Therefore, our overall analytical platform from unsupervised learning by MOFA+ to the validation of key‐drivers using the systems biological approaches, PBMCs, iPSC‐derived brain organoids, and human brain transcriptome can inform the mechanism of finding the rationales of clustering results from their unsupervised models as well as their novel potential subtypes.

### 2.10 MOFA+ Analysis Application to an Independent Cohort

Since the cohort for M‐IPAD and M‐TPAD models only included PiB^+^ participants, it was also necessary to confirm the discriminatory potential of the MOFA+ tool to identify CN+ and AD cases (regardless of their brain amyloid status, but according to the cognitive status). Therefore, we additionally performed MOFA+ analysis in an independent Chinese AD cohort (Figure S11, Table S2, Supporting Information), which comprised whole genome sequencing, proteomics, and blood biomarkers from 180 participants (*n =* 106 patients with AD, *n =* 74 CN+) (Figure S11a,b, Supporting Information). ROC curve analysis using Factor 2 showed a high AUC value (0.86) although it is an unsupervised multi‐omics method (Figure S11c, Supporting Information). Moreover, MOFA+ showed clear clusters and distinct proportion of CN+/AD ratio (Figure S11d–f, Supporting Information, left). Furthermore, one of the key biomarkers for AD, plasma neurofilament light chain, showed significant differences between the clusters both in all participants and AD patients only (Figure S11f,g, Supporting Information). Therefore, these findings confirm that MOFA+ can also be useful to discriminate AD from CN+ in an independent cohort, as well as to find potential clusters within the disease group.

## 3 Discussion

Current medications for AD are limited to acetylcholinesterase inhibitors or N‐methyl D‐aspartate antagonists. Although they alleviate clinical symptoms by decreasing cholinesterase activity or regulating glutamate in the brain, researchers do not yet fully understand how they work to treat AD. Moreover, since these drugs do not directly target the pathological molecules represented by A*β* peptides or aggregated tau, they cannot solve the fundamental problems that are relevant to unravel the main cause of the disease. Therefore, recent trials have focused on new drugs targeting specific molecules in the brain, such as aducanumab, solanezumab, and tau aggregation inhibitor, which have represented significant advances. However, it is still subject to debate whether it can be effective to every patient because AD is a multi‐factorial disease with various biological risk factors. Several researchers have suggested that the heterogeneity of AD exists; therefore, finding AD subtypes and developing personalized medications through integrated analytic approaches overseeing diverse pathways has become a need. Hence, we believe that our data, which reveals possible molecular drivers of AD heterogeneity or subtypes by using multiple datasets, is without doubt an extraordinarily important step for AD knowledge.

Fortunately, recent technological multi‐omics advances have paved the way for analytical methods that can integrate heterogeneous data sources.^[^ ^9^ ^]^ Herein, MOFA+, a computational method for unsupervised integration decomposing the sources of heterogeneity of data, enabled us to merge our multiple datatypes comprising several missing data and reveal novel putative molecular drivers of each possible AD subtype.^[^ ^11b^ ^]^ Although our miRNA or proteomics datasets had missing values, MOFA+ successfully performed the multi‐omics trainings with the reconstruction of the MOFs and revealed distinct clustering patterns in the M‐TPAD and M‐IPAD models (Figures 1 and 2). Using MOFA+, we obtained noteworthy results. First, the immune‐focused M‐IPAD model showed a higher clustering score (silhouette score, 0.64) than the M‐TPAD model (silhouette score, 0.41), which means that immunological changes may be involved in AD heterogeneity (Figure 2). Second, our clusters do not just rely on well‐known AD‐related factors, such as brain A*β* or cognitive scores (see Figures S4 and S5, Supporting Information). Third, various AD‐associated molecules such as bridging integrator 1 (BIN1), ATP binding cassette subfamily A member 7 (ABCA7), lipoprotein lipase (LPL), complement receptor 1 (CR1), vascular cell adhesion protein 1 (VCAM1), C‐reactive protein (CRP), galectin‐3 binding protein (LGALS3BP), galectin‐3, high‐density lipoprotein (HDL) cholesterol, and phosphoinositide 3‐kinase (PI3K) were selected as top‐rated targets for contributing to the clusters (Figures 3 and 4). However, we thought that further systems‐biological approaches for the M‐TPAD and M‐IPAD models were still needed for additional biological interpretation of the generated clusters.

For the biological interpretation, we set two main purposes: to find the relevant pathways involved in the identified clusters and to find a way to reveal the characteristics of each cluster (Figure 5). For the first goal, enriched pathway analysis was performed to reveal the key pathways of each cluster. Interestingly, several immune‐related pathways (immune systems, complement pathways, lipid metabolic process, complement activation, among others) were selected in the M‐IPAD model. Since dysregulation of lipid‐related pathways and altered cholesterol‐metabolism have fundamental roles in the progression of AD^[^ ^3^, ^17^ ^]^ and are highly associated with neuroinflammation in AD,^[^ ^18^ ^]^ we suggest our M‐IPAD model operated properly to understand biological implications consisting in the clustering results. Noteworthy, many pathways were also associated with autophagy pathways (e.g., autophagosome assembly, mTOR pathway, and PI3K‐Akt pathway) and AD‐related pathways (e.g., AGE‐RAGE pathway, histone deacetylation, and cholinergic synapse) both in the M‐TPAD and M‐IPAD models. Furthermore, each cluster showed distinct differences although they were all comprised PiB+ participants without any differences in their cognitive functions, age, sex, ApoE type, and their brain status (see Figures S4 and S5, Supporting Information). Thus, through our enriched pathway analysis, each cluster was able to be distinguished because of their differently enriched pathways. Next, for the second goal, multiple clustering analysis (MCA), and key driver analysis were performed, revealing the key driver nodes within the network model,^[^ ^14^ ^]^ Interestingly, the key‐driver node analysis along with our longitudinal analysis proposed that autophagy is the main cause of the association between longitudinal changes of the brain and our clustering (Figure 6, see Table S9, Supporting Information). Even if two years is not a long time for a longitudinal study, we confirmed that there have been many changes in the condition of participants for 2 years (cognitive decline was observed in 63.5% of patients, PiB increase was observed in 77.9% of patients, FDG decrease was observed in 89.8% of patients, Hva decrease in 73.8% of patients, and clinical dementia rating changes in 26.8% of patients). We believe that this clinical or pathological deterioration for two years made us interpret the association between the clustering information and the condition of participants. Also, since i) autophagy‐related pathways are highly associated with Hva changes, glucose metabolism, and broad ranges of neurodegeneration, ii) autophagy is one of the major links between the periphery and the brain, iii) both peripheral and central autophagic regulatory systems are highly interlinked with the progression of AD (Figure 7), and iv) clusters within the AD patients are also clearly shown in another cohort (Figure 8), we suggested that our interpretation provides reasonable evidence for the appropriate MOFA+ clustering. Hence, we concluded that characterization of the subtypes within AD patients was completed by convergence of multi‐omics analysis, systems‐biological approaches, and biological validation with PBMCs, iPSC‐derived organoids, and human post‐mortem brain samples. Although several studies have recently examined the multi‐modality of AD pathology, our analytical platform has obvious differentiation strategies because i) all datasets for the multi‐omics analyses were generated autonomously, ii) we adopted system‐biological analyses with HENA AD network, KDA, and MCA methods to narrow‐down the key‐drivers, iii) we performed a longitudinal analysis to understand the informative clusters in‐depth, and iv) we utilized different types of biological samples to validate our key‐drivers such as PBMCs, iPSC‐derived cerebral organoids, microglia, and human post‐mortem brain samples.

In summary, this study revealed distinct clusters in patients with AD, both in the general (M‐TPAD) and immunological (M‐IPAD) models. Although a few studies on multi‐omics data or omics‐based subtyping trials exist, our analyses provide differentiated strategies. Neff et al.^[^ ^8^ ^]^ only used RNA sequencing data from two different public cohorts (ROSMAP and MSBB), whereas we used four different multi‐layers (targeted‐sequencing, miRNA transcriptome, proteomics, blood‐based biomarkers) that were generated autonomously. Furthermore, Clark et al.^[^ ^19^ ^]^ utilized MOFA software in R to combine their multi‐modal datasets in AD but were restricted to the MOFA software, whereas i) we adopted systems‐biological analyses with HENA AD network, KDA, and MCA methods to narrow‐down the key‐drivers, ii) performed a longitudinal analysis to elucidate the informative clusters, and iii) utilized different types of biological samples to validate our key‐drivers such as PBMCs, iPSC‐derived cerebral organoids, and human post‐mortem brain samples. Furthermore, our systems‐biological approaches identified that there are significant associations between the top‐rated targets and the enriched pathways or meanings of each cluster in both models. Nevertheless, further studies with more focus on testing candidate drugs (e.g., treatment with drugs to cerebral organoids or clinical trials) should be performed to develop precision medicine therapies for AD. Moreover, further validation using an independent cohort with similar multi‐modal datasets is needed. Despite these limitations, we believe that this study provides a powerful platform for materializing precision medicine treatments for AD relying on the convergence of multi‐omics analysis, network modeling by a systems‐biological approach, a longitudinal cohort analysis, and validations using PBMCs, iPSC‐derived cerebral organoids, and human post‐mortem brain samples.

## 4 Experimental Section

### Recruitment of Participants and Brain Imaging

A total of 170 patients with Pittsburgh compound B‐positron emission tomography (PiB‐PET) positive (brain amyloid positive), who participated in the Korean Brain Aging Study for the Early Diagnosis and Prediction of AD (KBASE), were included in the study (see Tables S1–S4, Supporting Information). All participants underwent comprehensive evaluations and multi‐modal brain imaging, such as PiB‐PET, ^18^F fluorodeoxyglucose (FDG)‐PET, and magnetic resonance imaging (MRI). All of the methods related to the assessment of the patients and their imaging data were described in the previous study, in which the characteristics of the recruited participants were further summarized.^[^ ^20^ ^]^ Briefly, for surrogate markers of in vivo neuropathological changes in AD, standardized uptake value ratio of accumulated PiB was quantified in the cortical region‐of‐interests. Moreover, the standardized uptake value ratio of FDG‐PET was used to measure cerebral glucose metabolism in the brain of the participants, which was related to functional deficits in AD.^[^ ^21^ ^]^ T1 MRI was used for Hva and cortical thickness (Dickerson) assessments based on previous studies.^[^ ^22^ ^]^

### Multimodal Generation

Diverse experimental and analytic tools were used prior to the machine learning processes, using human blood‐derived biopsied specimens such as PBMC, DNA, plasma, serum, and miRNA from 170 patients with cerebral amyloid pathology. Detailed methods for the generation of each dataset (genetics, transcriptomics, proteomics, and blood‐based biomarkers) are presented in Supporting Information.

### Unsupervised Integration of Multi‐Omics Datasets

For the unsupervised integration of datasets (Please see details in Supporting Information), Multi‐Omics Factor Analysis V2 (MOFA+ version 1.1; with R version 4.0.0 and R Studio version 1.1.456) was applied, which was developed for decomposing the sources of heterogeneity in multi‐omics datasets, to high‐dimensional multi‐omics profiles collected from PiB+ patients‐derived peripheral blood samples.^[^ ^11^ ^]^ All analyses in MOFA+ were performed in accordance with MOFA+ vignette, which can be freely downloaded at https://github.com/bioFAM/MOFA2.

### Statistical Analysis for the Downstream Analyses beyond MOFA+

For clustering, standardized criteria were set and applied to the trained MOFs (Figure 2). The elbow plot and average silhouette analysis were performed using ggplot2 R package (version 3.3.3), to determine the optimal number of clusters and factor‐combination.^[^ ^23^ ^]^ Please see details in Supporting Information.

### Enriched Pathway Analysis, Multiple Clustering Analysis (MCA), and Key‐Driver Analysis (KDA)

Targets that showed a significant increase or decrease rate (threshold > 0.5 or < −0.5, respectively) compared with the average value of the whole cohort were used as input to reveal important pathways of each cluster. Six public databases were used (KEGG_2019_human, GO_molecular_function_2018, GO_cellular_component_2018, GO_biological_process_2018, BioCarta_2016, Reactome_2016). MCA followed by KDA was performed with a network model from a heterogeneous network‐based dataset for AD (HENA) and the analysis for multiscale clustering of geometrical network (MEGENA).^[^ ^14^, ^24^ ^]^ Please see details in Supporting Information.

### Generation of iPSC‐Derived Brain Organoids, Microglia, and Brain Assembloids

Cerebral organoids were generated from human PBMC‐derived iPSC lines in accordance with the same method from the previous paper.^[^ ^3f^ ^]^ The twelve iPSC lines in that paper were also used for this project. Briefly, embryoid bodies (EBs) were formed in AggreWell EB formation Medium (Stemcell Technologies; Vancouver, Canada). The EBs were grown in iPSC‐culture medium containing SMAD inhibitors, dorsomorphin (Merck, NJ, USA), and SB‐431542 (TOCRIS, Bristol, UK) and transferred to 96 well ultra‐low‐attachment plates (Corning, NY, USA) on day 6. From day 7, organoids were cultured in neurobasal medium with a variety of supplements. For experiments, days in vitro (DIV) 70 organoids or DIV100 organoids were used for western blot, immunohistochemistry, and generation of brain assembloids. Next, iPSC‐derived microglia were generated according to the previous paper.^[^ ^25^ ^]^ In brief, to generate primitive hematopoietic progenitor cells (HPCs), small aggregates of iPSCs were cultured in HPC medium (Stemcell technologies) for 12 days. The round‐shaped non‐adherent HPCs were transferred into new iPSC‐microglia medium containing 100 ng mL^−1^ IL‐34, 50 ng mL^−1^ TGF*β*1, and 25 ng mL^−1^ M‐CSF (PeproTech, Seoul, Korea) to induce differentiation of microglia for 25 days. On DIV 25, cells were resuspended in iPSC‐microglia medium and further supplemented with 100 ng mL^−1^ CD200 (Novoprotein, CA, USA) and 100 ng mL^−1^ CX3CL1 (PeproTech). On DIV 31, cells were used for the generation of brain assembloids. For the generation of brain assembloids, DIV 100 organoids and DIV 31 microglia (2×10^5^ cells) were incubated together in neurobasal medium with five cytokine cocktails (IL‐34, TGF*β*1, M‐CSF, CD200, and CX3CL1) for 7 days. The generated brain assembloids were used for immunohistochemistry and analyzed with IMARIS software (Bitplane, Zurich, Switzerland).

### Western Blotting and RNA Sequencing using Human PBMCs, iPSC‐Derived Brain Organoids, and Human Post‐Mortem Brain Samples

For validation I (Figure 7), the western blot analysis was performed to quantify autophagy‐related molecules, according to the previous study.^[^ ^26^ ^]^ PBMC or brain organoid samples were briefly lysed with RIPA buffer containing protease inhibitor cocktail. Total proteins were extracted and quantified by BCA assay. Equal quantities of the cell lysate (protein concentration) were loaded on each well of 4–12% Nupage gels (Thermo Fisher Scientific, MA, USA) and separated. The gels were transferred to a polyvinylidene difluoride (PVDF) membrane, which was blocked with a blocking solution for 1 h. Subsequently, the membrane was washed for 30 min and incubated with primary antibodies overnight at 4 ℃. The following day, the membrane was washed again and incubated with secondary antibodies for 1 h. The protein bands were visualized with a bio‐imaging analyzer (AI600, GE Healthcare, IL, USA) and quantified with a Multi‐Gauge Software (Fujifilm Corporation, Tokyo, Japan). Full blots are shown in Figures S12 and S13, Supporting Information. For the gene expression of autophagy‐related molecules in the iPSC‐derived brain organoids, the previous RNA sequencing data with a telomerase inhibitor (MST‐312; Sigma‐Aldrich, MO, USA) were re‐analyzed. The sequencing data were already available at NCBI under SRA accession number PRJNA678865. For the detailed methods of the RNA sequencing, please refer to the previously published paper.^[^ ^3f^ ^]^ For validation II (Figure 8), a public transcriptome database was utilized with the human post‐mortem brains (*n =* 78) from the NCBI GEO database (GSE109887). Clustering analysis was performed using pheatmap and ggplot2 packages in the R software. DEGs between the clusters were analyzed using GEO2R (https://www.ncbi.nih.gov/geo/geo2r).

### Ethical Approval

Please see details of the methods for recruitment of participants and brain imaging in Supporting Information. The Institutional Review Board of the Seoul National University Hospital (South Korea) approved this study (E‐2009‐120‐1159). Participants or their legal guardians provided written informed consent.

### Statistical Analysis

All input data were pre‐processed and quality‐controlled according to the detailed steps described in Figures S1 and S2, Supporting Information. Outlier values were excluded by ROUT (Q = 1%) method and clustering analysis. All input values were transformed within the range from 0 to 1 (Min‐Max normalization method). Data were presented as mean ± SEM, and p‐values were calculated by independent *t*‐test, ANOVA post‐hoc test, and chi‐square test. Sample size for each statistical analysis was presented in appropriate Figure legends and described in detail in the results section. All statistical analyses beyond R software were conducted by Medcalc software (ver. 20.009; Ostend, Belgium) and Graphpad Prism 8 (San Diego, CA, USA).

## Conflict of Interest

The authors declare no conflict of interest.

## Author Contributions

N.B.‐T. and S.‐Y.J. contributed equally to this work. Conceptualization: J.‐C.P., I.M.‐J., and J.H. Investigation: J.‐C.P., H.J.K., and H.L. Methodology & Visualization: J.‐C.P., N.B.T., S.‐Y.J., K.Y.M., H.J.K., J.S., S.‐H.H., H.J., M.S.B., J.W.H., and D.Y. Resources: J.‐C.P., K.‐H.C., M.C., D.H., S.‐W.L., D.Y.L., M.S.B., and D.Y. Writing‐ original draft: J.‐C.P. Writing‐ review & editing: J.‐C.P., I.M.‐J., and J.H.

## Supporting information

Supporting Information

Supporting Information

## Data Availability

The data that support the findings of this study are openly available in Github at https://github.com/jcparkgithub/multiomics_for_mtpad_mipad, reference number 0.
