# Supplementary material for: Multi‐Omics‐Based Autophagy‐Related Untypical Subtypes in Patients with Cerebral Amyloid Pathology
Source: Adv Sci (Weinh). 2022 Jun 13;9(23):2201212. doi: 10.1002/advs.202201212 (PMC9376815; doi:10.1002/advs.202201212)
Supplement: Supplementary file 1 — Supporting Information [file ADVS-9-2201212-s002.pdf]

ADVANCED SCIENCE
Open Access

Supporting Information

for *Adv. Sci.*, DOI 10.1002/advs.202201212

Multi-Omics-Based Autophagy-Related Untypical Subtypes in Patients with Cerebral Amyloid Pathology

Jong-Chan Park, Natalia Barahona-Torres, So-Yeong Jang, Kin Y. Mok, Haeng Jun Kim, Sun-Ho Han, Kwang-Hyun Cho, Xiaopu Zhou, Amy K. Y. Fu, Nancy Y. Ip, Jieun Seo, Murim Choi, Hyobin Jeong, Daehee Hwang, Dong Young Lee, Min Soo Byun, Dahyun Yi, Jong Won Han, Inhee Mook-Jung and John Hardy**

Supporting Information

Title: Multi-omics-based autophagy-related untypical subtypes in patients with cerebral amyloid pathology

Authors: Jong-Chan Park[1,2,3,4], Natalia Barahona-Torres[1†], So-Young Jang[5†], Kin Y. Mok[1], Haeng Jun Kim[2,4], Sun-Ho Han[2,3,4], Kwang-Hyun Cho[5], Xiaopu Zhou[6,7,8], Amy K. Y. Fu[6,7,8], Nancy Y. Ip[6,7,8], Jieun Seo[9], Murim Choi[2], Hyobin Jeong[10], Daehee Hwang[11], Dong Young Lee[12,13,14], Min Soo Byun[15], Dahyun Yi[16], Jong Won Han[2], Inhee Mook-Jung[2,3,4]*, and John Hardy[1]*

- **Supporting figures**

Figure S1. Detailed multi-omics approaches of this study.

Figure S2. Detailed process for QC and normalization of datasets for the M-TPAD and M-IPAD model.

Figure S3. Elbow-plots, silhouette analysis, and clustering graphs for every combination of possible MOFs.

Figure S4. Comparison of AD-related known-factors between the clusters.

Figure S5. No interference of the AD-related known-factors on the M-TPAD/IPAD model.

Figure S6. Correlation between factor values and the highest weighted targets from the M-TPAD and M-IPAD models and ROC curve analyses using the highest weighted targets.

Figure S7. Target-association network models from each MOFs and their functional partners.

Figure S8. Target-association network models (context-specific PPI) from each MOFs and their functional partners.

Figure S9. Longitudinal changes in cognition scores and brain-imaging biomarkers for two years.

Figure S10. Comparison of the levels of autophagy-related markers in human PBMC samples between ApoE ε4 carriers vs ε4 non-carriers (only CN PiB-PET negative group included) and generation of brain organoids & assembloids.

Figure S11. Multi-omics analysis in a Chinese Alzheimer's disease cohort.

Figure S12. Full blots with a size marker for autophagy-related molecules in PBMC samples (related to Figure 7).

Figure S13. Full blots with a size marker (Novex size marker from Thermo scientific, cat: 57318) for autophagy-related molecules in CRISPR-Cas9-based ApoE isotype (ε3/ε3, parental line; ε4/ε4, isogenic line) brain organoid samples (related to Figure 7).

- **Supporting tables**

Table S1. Demographic data of the participants of the study (n = 170; PiB+ participants).

Table S2. Demographic characteristics of the Chinese AD cohort (related to Figure S11).

Table S3. Demographic characteristics of PBMC validation cohort (n = 120).

Table S4. Demographic characteristics of iPSC-derived brain organoid cohort (n = 10).

Table S5. Demographic data of the M-TPAD clusters.

Table S6. Demographic data of the M-IPAD clusters.

Table S7. Details of ROC curve analyses (related to Figure 2).

Table S8. Details of ROC curve analysis (related to Figure S6).

Table S9. List of references related to autophagy pathways (related to Figure 6F).

- **Supporting methods**

- **Supporting references**

Supporting figures

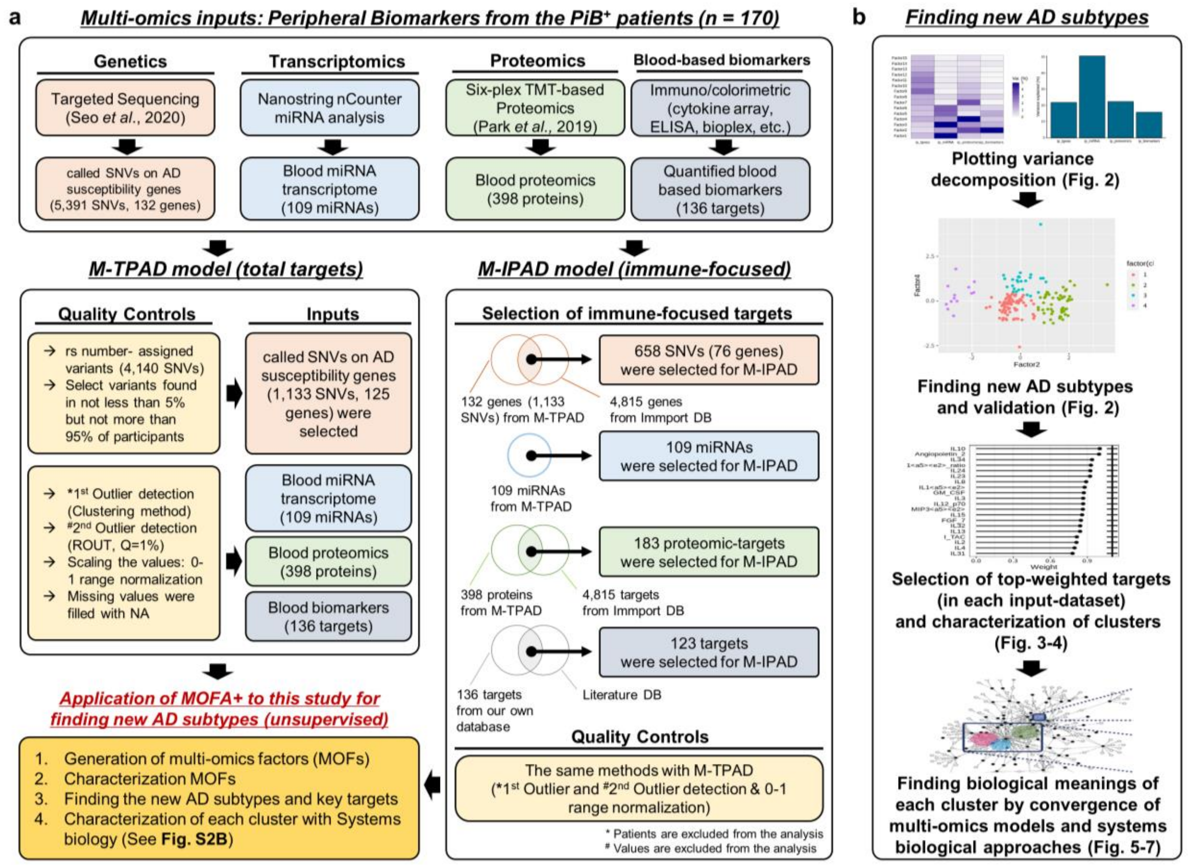

Figure S1. Detailed multi-omics approaches of this study. (a) Four input-datasets (layers) were included in the multi-omics analysis. The appropriate quality-controls (QCs) and normalization for each dataset were performed (see details in Figure S1). The immune-focused model (M-IPAD) was generated independently, as well as the total-target model (M-TPAD). Through the unsupervised integration using the MOFA+ in R software, new subtypes of AD were discovered. **(b)** The process of down-stream analyses followed by systems-biological approaches for the characterization of Alzheimer's disease (AD) subtypes. The learnt factors were used for the downstream analyses and the generated clusters were characterized. The top-weighted targets (gene, miRNA, or proteins) from each layer were identified and used for the systems biological analyses. After the multi-omics analyses, final validation was performed using human peripheral mononuclear cells (PBMCs) and induced-pluripotent stem cells (iPSCs)-derived brain organoids. **Abbreviations:** M-IPAD, multi-omics-based immune profiling for AD; M-TPAD, multi-omics-based total profiling for AD; MOF, multi-omics factor; MOFA, multi-omics factor analysis; SNV, single nucleotide variant.

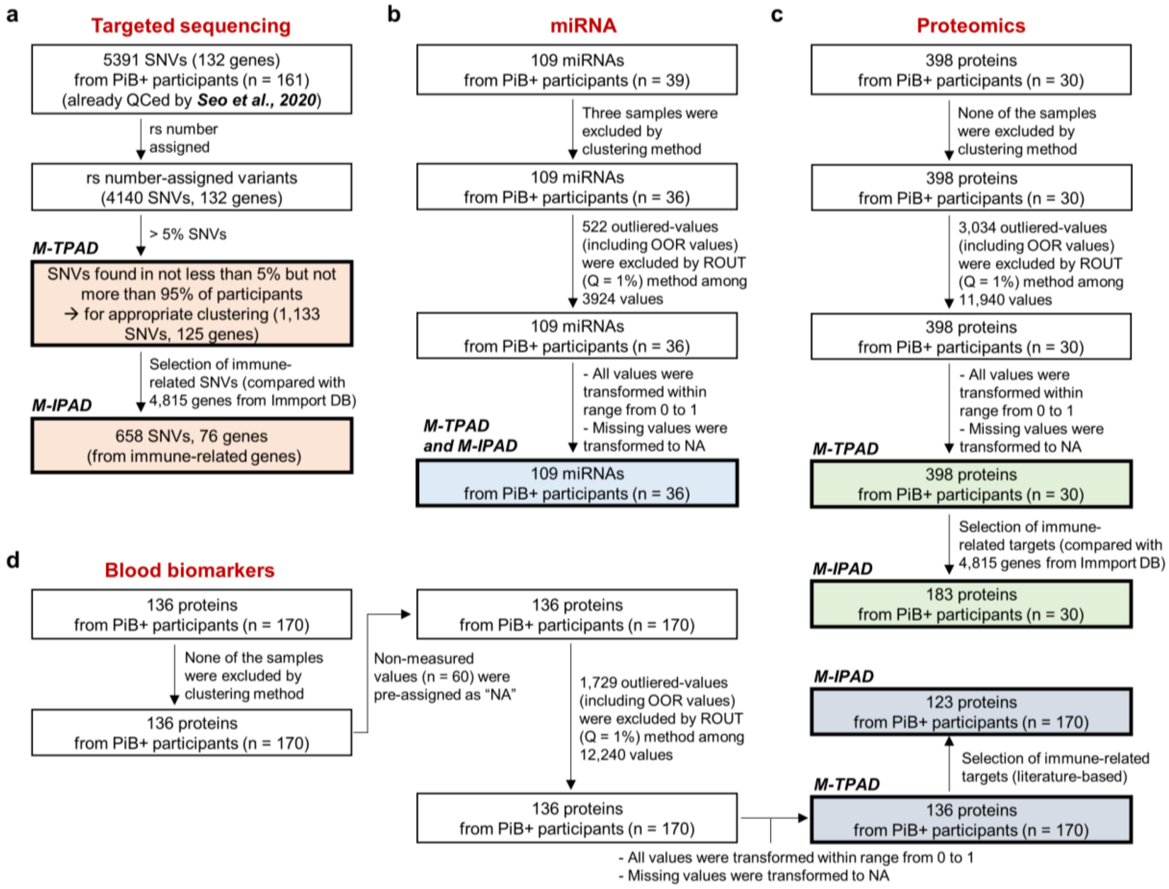

Figure S2. Detailed process for QC and normalization of datasets for the M-TPAD and M-IPAD model. (a) Targeted Sequencing, **(b)** miRNA, **(c)** proteomics, and **(d)** blood-based biomarkers. **Abbreviations:** DB, database; n, number of patients; NA, non-applicable; OOR, out of range; QC, quality-control; SNV, single nucleotide variants; ROUT, robust regression and outlier removal.

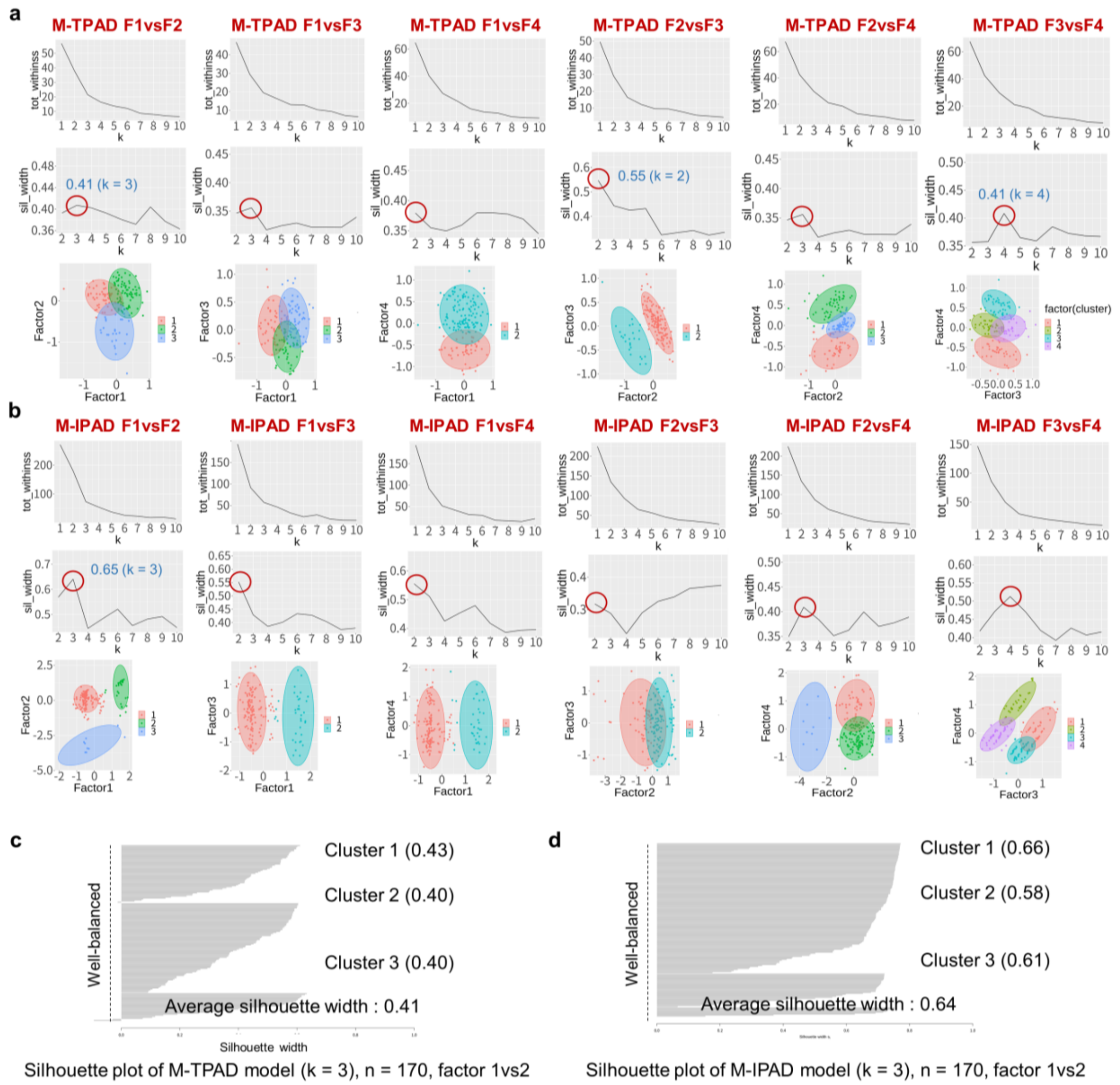

Figure S3. Elbow-plots, silhouette analysis, and clustering graphs for every combination of possible MOFs (a) Visualization of elbow-plots, silhouette curves, and samples according to each combination of factors for the M-TPAD model by *ggplot2* in R software. **(b)** Visualization of elbow plots, silhouette curves, and samples according to each combination of factors for the M-IPAD model. For both models, k value was considered within the range of $2 \leq k \leq 5$. Red circles show the highest point of silhouette scores of each graph. Shaded circles show clusters that are determined by the k-medoids method (k, the highest scores from each silhouette analysis). **(c)** Silhouette scores of individual patients in the M-TPAD model (k = 3, n = 170, factor 1 vs. factor 2). All clusters had similar and well-balanced (± 10% from total average) silhouette scores (average, 0.41), although they were relatively lower than those of the M-IPAD model. **(d)** Silhouette scores of individual patients in the M-IPAD model (k = 3, n = 170, factor 1 vs. factor 2). All clusters had similar and well-balanced (± 10% from total average) silhouette scores (average, 0.64). **Abbreviations:** MOFs, multi-omics factors; sil_width, silhouette width; tot_withinss, total within-cluster sum of squares.

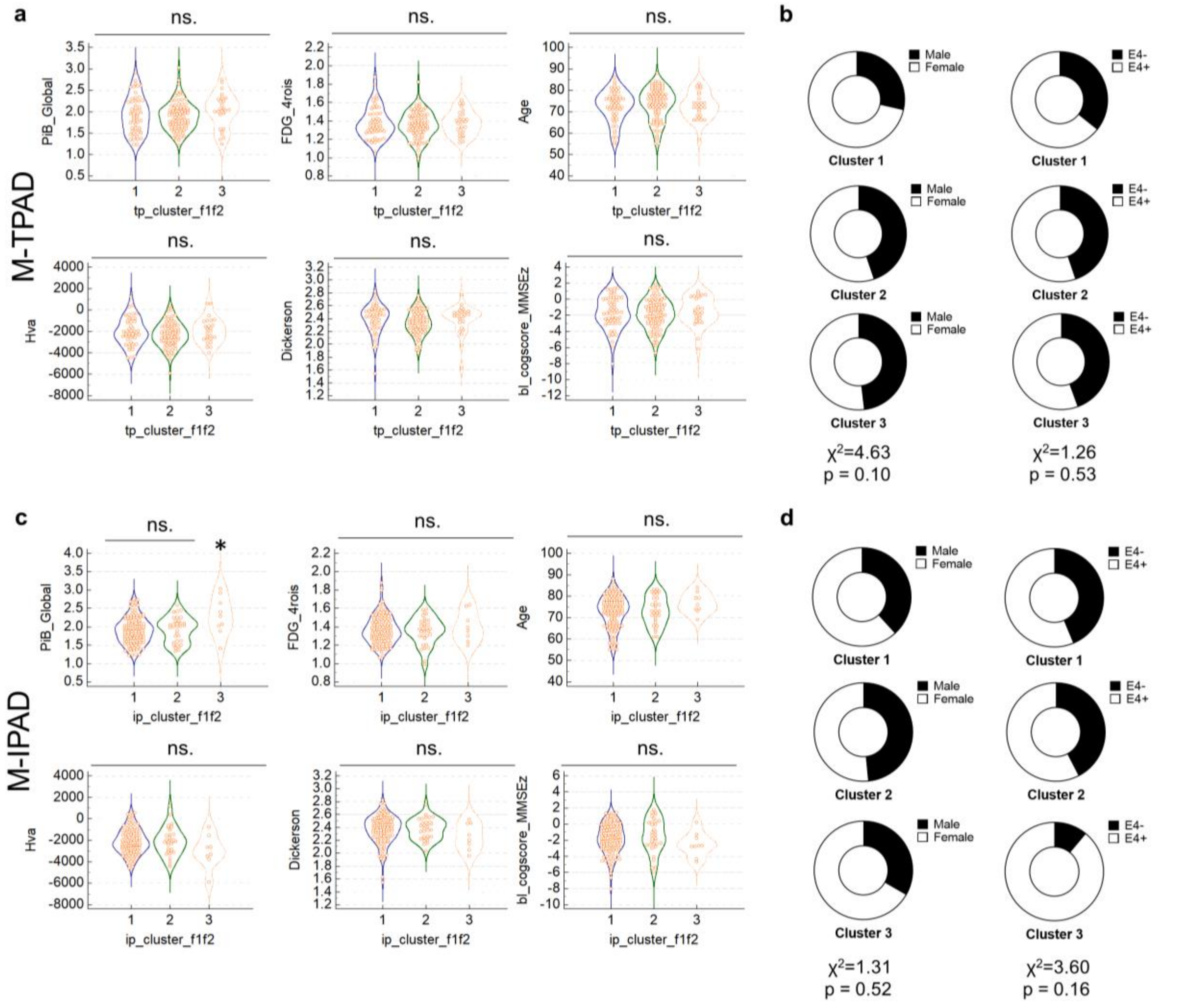

Figure S4. Comparison of AD-related known-factors between the clusters (a) Comparison of known factors related to AD between the clusters of the M-TPAD model. P-values were obtained from ANOVA with post-hoc test. **(b)** Chi-square test between the clusters for sex and apolipoprotein E4 allele for the M-TPAD model. **(c)** Comparison of known factors related to AD between the clusters of the M-IPAD model. P-values were obtained from ANOVA with post-hoc test. *$p < 0.05$. **(d)** Chi-square test between the clusters for sex and apolipoprotein E4 allele for the M-IPAD model. **Abbreviations:** χ^2, chi-square; Dickerson, cortical thickness; E4, Apolipoprotein E ε4; FDG_4rois, flurodeoxyglucose-PET SUVR values; Hva, hippocampal volume; ip_cluster_f1f2, clusters by the M-IPAD model; MMSEz, mini-mental status exam with correction for age, sex, and education; PiB_Global, Pittsburgh compound B-positron emission tomography SUVR values for global region; SUVR, standardized uptake value ratio; tp_cluster_f1f2, clusters by the M-TPAD model.

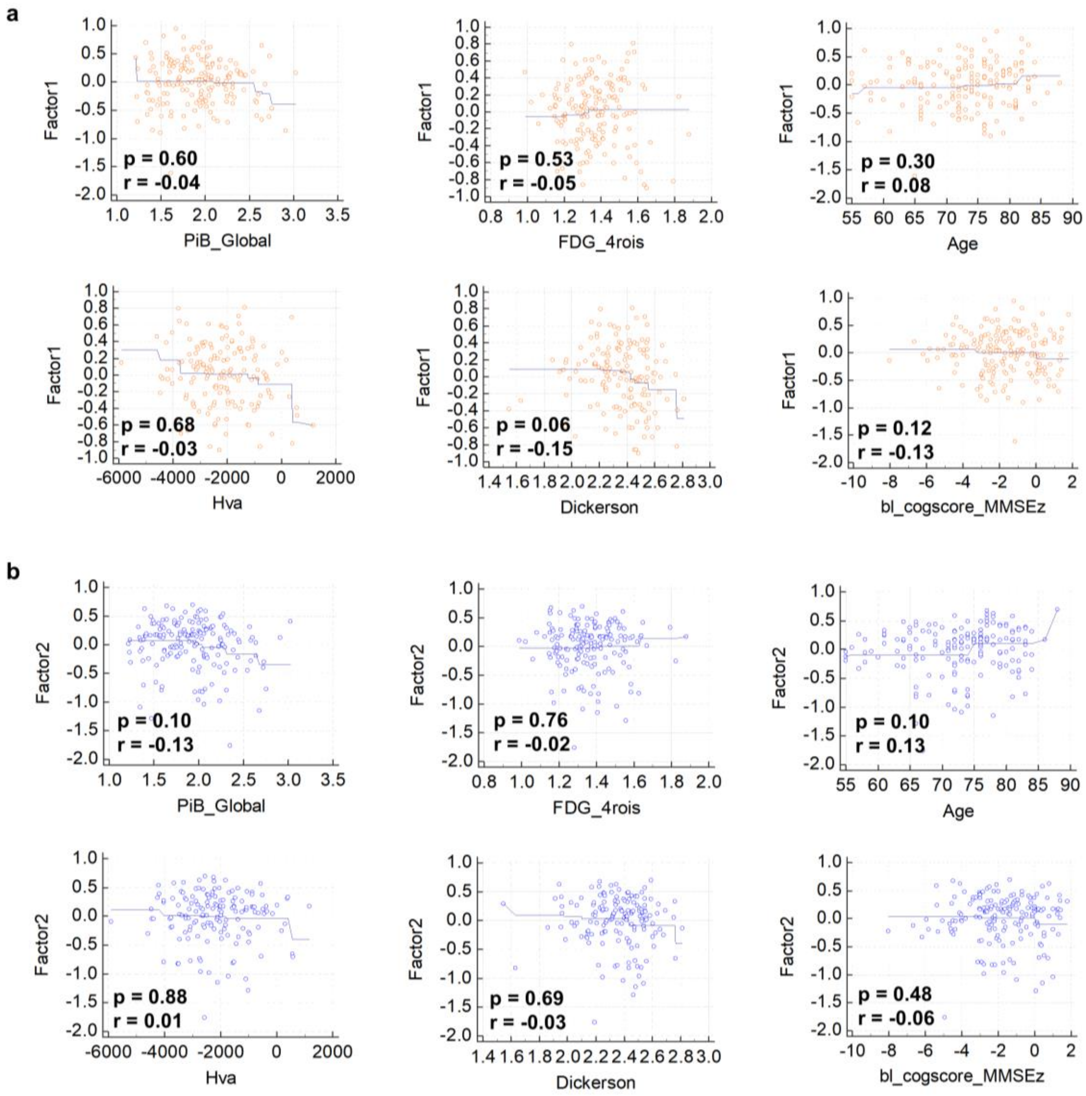

Figure S5-1. No interference of the AD-related known-factors on the M-TPAD model (a) No significant correlations between known-factors (PiB-PET, FDG-PET, hippocampal volume, cortical thickness, MMSE z-score, age) and factor 1 values were observed. P-values and r-values were obtained by Pearson's correlation analysis. **(b)** Non-significant correlations between known-factors and factor 2 values were observed. P-values and r-values were obtained by Pearson's correlation analysis. **Abbreviations:** Dickerson, cortical thickness; FDG_4rois, flurodeoxyglucose-PET SUVR values; Hva, hippocampal volume; MMSEz, mini-mental status exam z-score with correction for age, sex, and education; PiB_Global, Pittsburgh compound B-positron emission tomography SUVR values for global region; SUVR, standardized uptake value ratio.

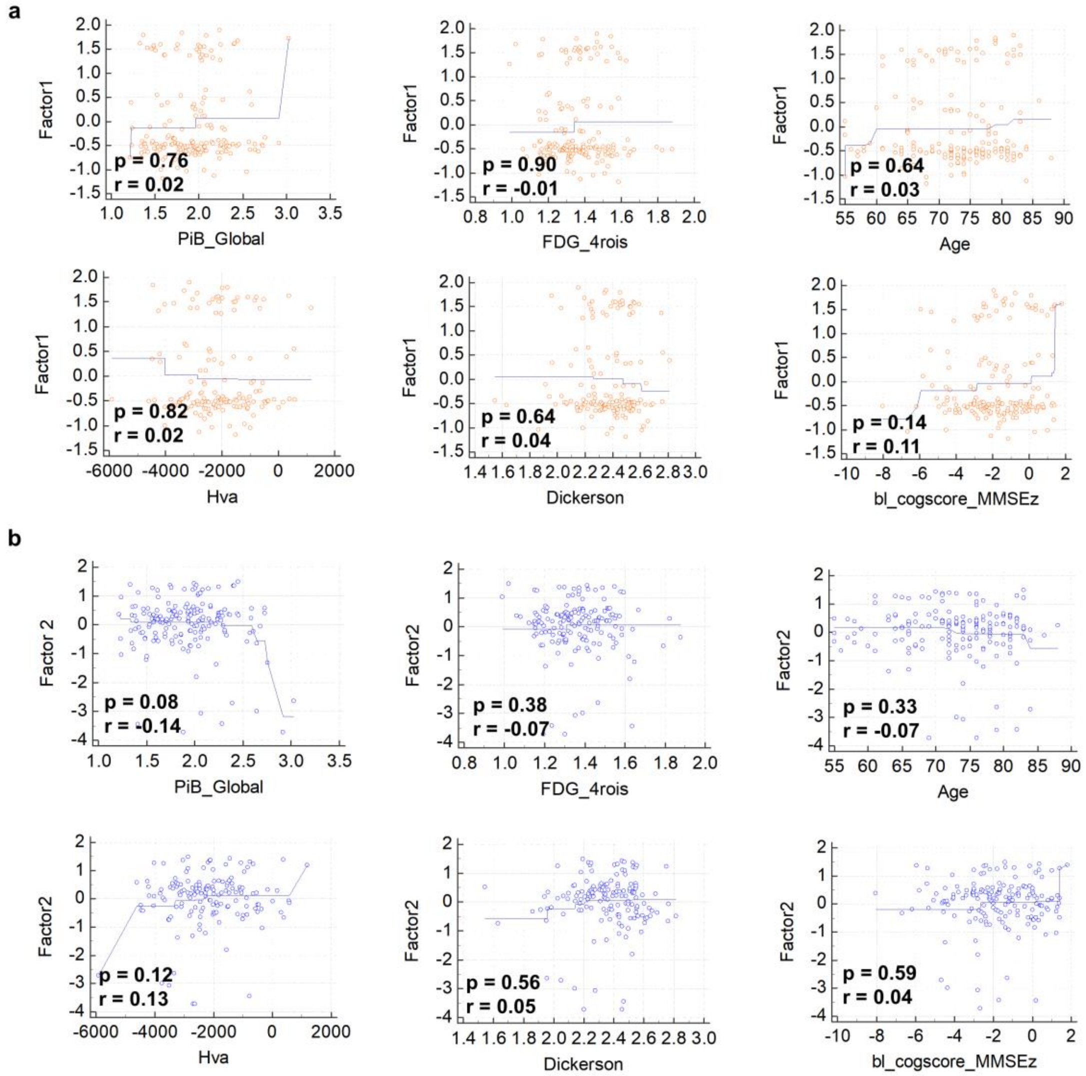

Figure S5-2. No interference of the AD-related known-factors on the M-IPAD model (a) No significant correlations between known-factors (PiB-PET, FDG-PET, hippocampal volume, cortical thickness, MMSE z-score, age) and factor 1 values were observed. P-values and r-values were obtained by Pearson's correlation analysis. **(b)** Non-significant correlations between known-factors and factor 2 values were observed. P-values and r-values were obtained by Pearson's correlation analysis. **Abbreviations:** Dickerson, cortical thickness; FDG_4rois, flurodeoxyglucose-PET SUVR values; Hva, hippocampal volume; MMSEz, mini-mental status exam z-score with correction for age, sex, and education; PiB_Global, Pittsburgh compound B-positron emission tomography SUVR values for global region; SUVR, standardized uptake value ratio.

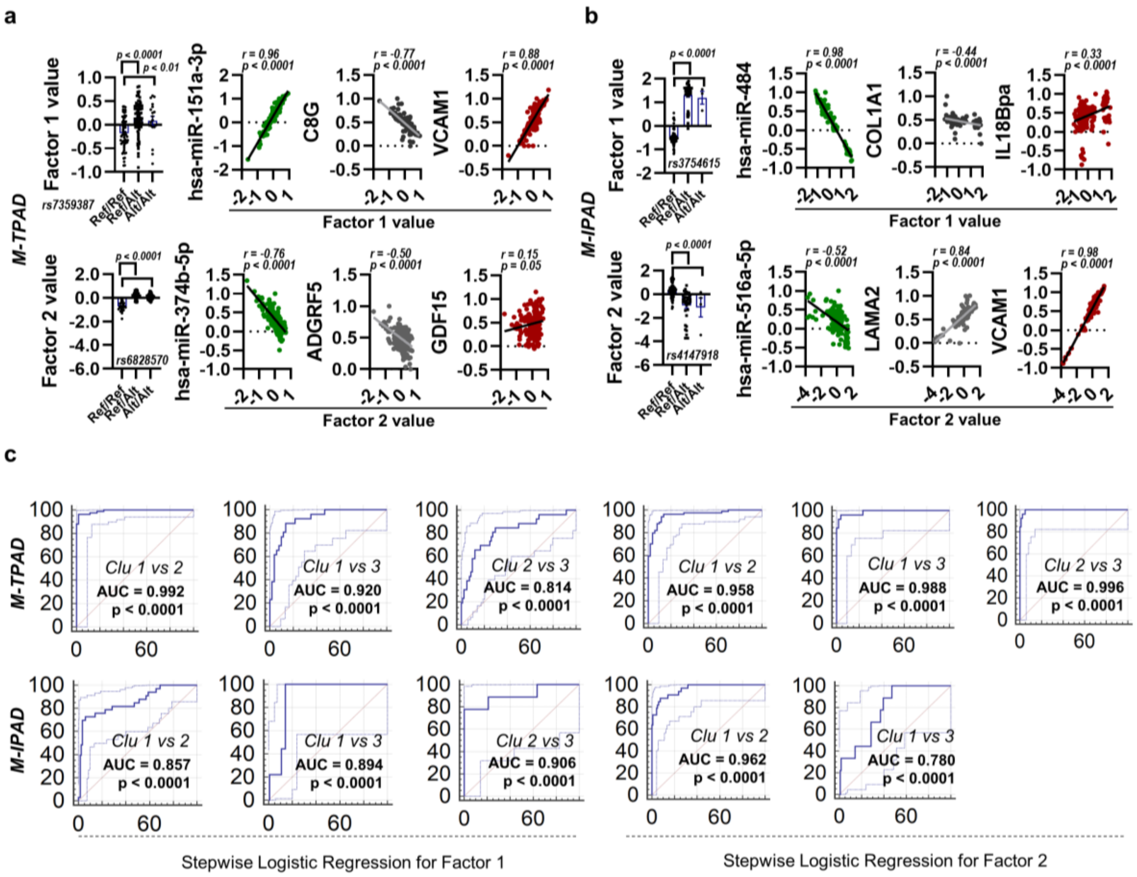

Figure S6. Correlation between factor values and the highest weighted targets from the M-TPAD and M-IPAD models and ROC curve analyses using the highest weighted targets (a) Significant correlations between the factor values and the highest weighted targets for the M-TPAD model. **(b)** Significant correlations between the factor values and the highest weighted targets for the M-IPAD model. For comparison SNV alleles, *p*-values were obtained by ANOVA with Tukey's post-hoc test. For other datasets, *p*- and r-values were obtained by Pearson's correlation analysis. **(c)** Stepwise logistic regression analyses followed by ROC curve analyses for the M-TPAD model. For factor 1, rs7359387, has-miR-151a-3p, C8G, and VCAM1 were selected for the logistic regression analysis. For factor 2, rs6828570, has-miR-374b-5p, ADGRF5, and GDF15 were selected for the logistic regression analysis. **(D)** Stepwise logistic regression analyses followed by ROC curve analyses for the M-IPAD model. For factor 1, rs3754615, has-miR-484, COL1A1, and IL18Bpa were selected for the logistic regression analysis. For factor 2, rs4147918, has-miR-516a-5p, LAMA2, and VCAM1 were selected for the logistic regression analysis. **Abbreviations:** Alt, alternative sequence; Ref, reference sequence.

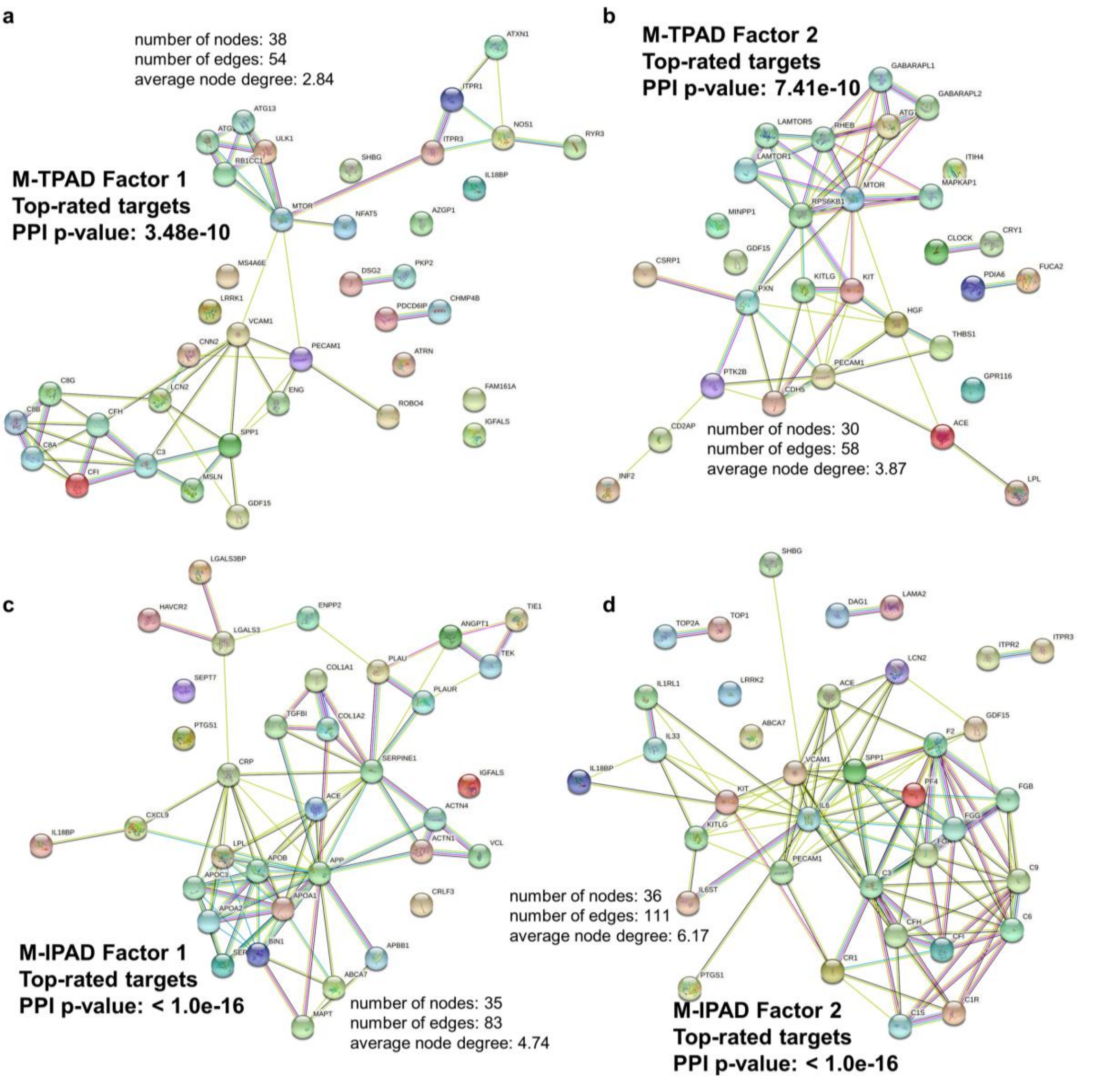

Figure S7. Target-association network models from each MOFs and their functional partners Results from the STRING database (functional protein-protein association analysis) using the top-rated targets of the **(a)** targets from M-TPAD Factor 1, **(b)** targets from M-TPAD Factor 2, **(c)** targets from M-IPAD Factor 1, and **(d)** targets from M-IPAD Factor 2. Several proteins (RB1CC1, ATG101, CFH, ATG13, PKP2, C3, C8A, CHMP4B, MTOR, and C8B for M-TPAD Factor 1; CRY1, GABARAPL2, RPS6KB1, KITLG, LAMTOR5, RHEB, MAPKAP1, GABARAPL1, PXN, and LAMTOR1 for M-TPAD Factor 2; VCL, SERPINE1, APOC3, APOB, ACTN4, COL1A2, PLAUR, APOA2, TEK, and APB1 for M-IPAD Factor 1; KITLG, C3, FGB, F2, FGG, IL33, C1S, IL6, TOP2A, and DAG1 for M-IPAD Factor 2) were used as predicted functional partners in the network model. Colored nodes, query proteins and first shell of interactors; white nodes, second shell of interactors; filled nodes, some 3D structure is known or predicted; edges, protein-protein associations. **Abbreviations:** PPI, protein-protein interaction.

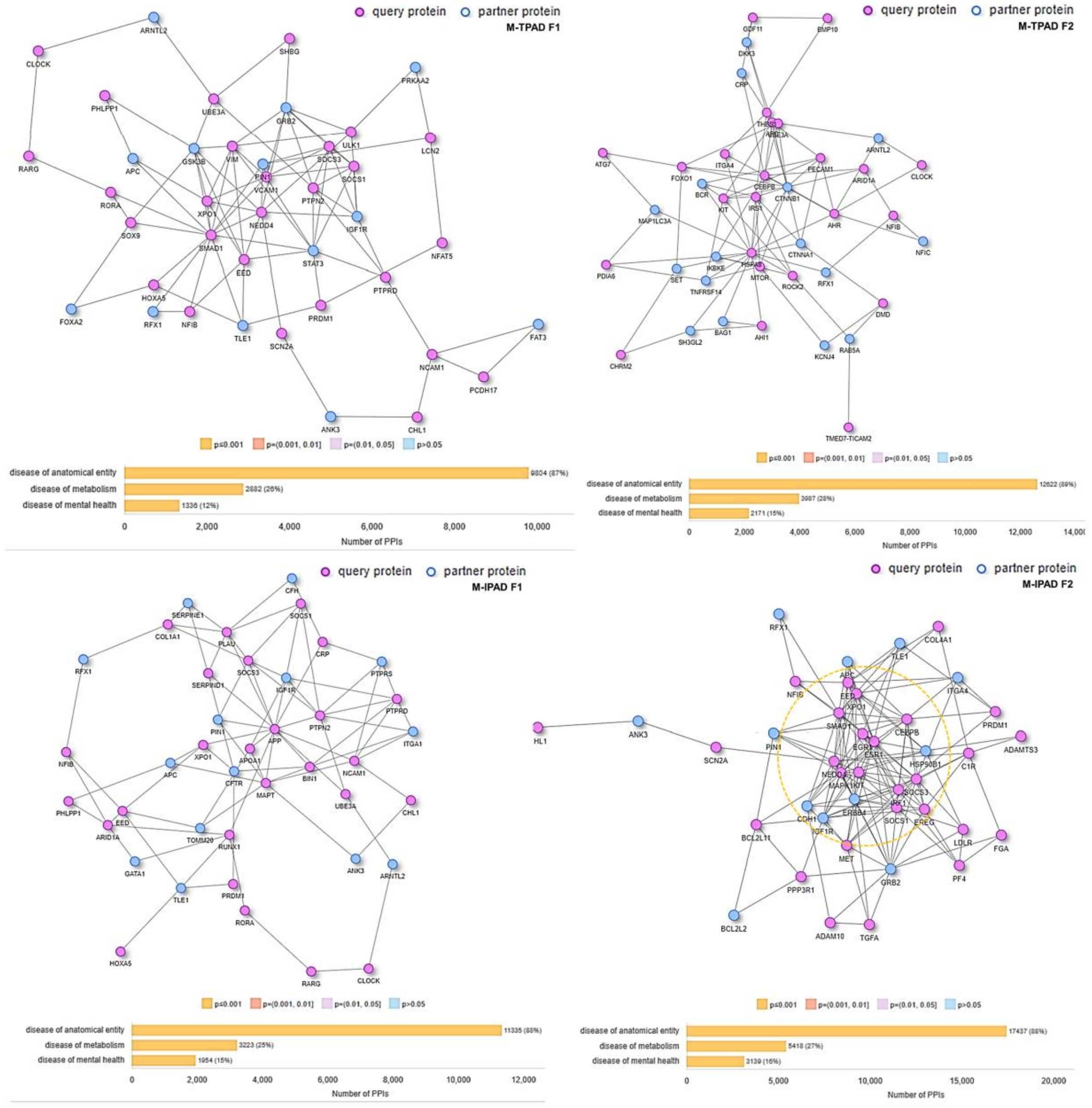

Figure S8. Target-association network models (context-specific PPI network) from each MOF and their functional partners Results from the integrated interactions database (IID) (context-specific functional protein-protein association analysis) using the top-rated targets of the M-TPAD and M-IPAD model. Pink coloured nodes, query proteins; blue nodes, partner proteins; edges, protein-protein associations; yellow circle, the most densely populated PPIs. **Abbreviations:** IID, integrated interactions database; PPI, protein-protein interaction.

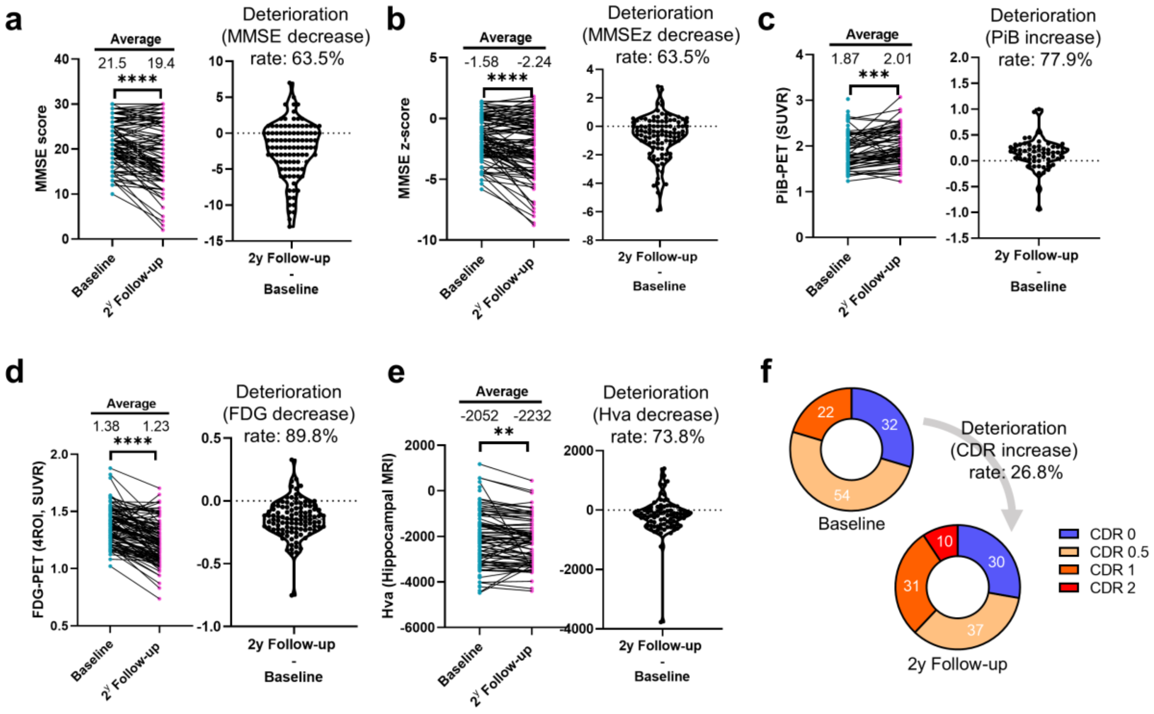

Figure S9. Longitudinal changes in cognition scores and brain-imaging biomarkers for two years. Cognition scores (mini-mental state examination scores or clinical dementia rating) or brain imaging biomarkers (PiB-PET, FDG-PET, and hippocampal volume changes) were significantly different between baseline cohort and 2-year follow-up cohort (*p < 0.05, **p < 0.01, ***p < 0.001, and ****p < 0.0001; two-tailed paired *t*-test).

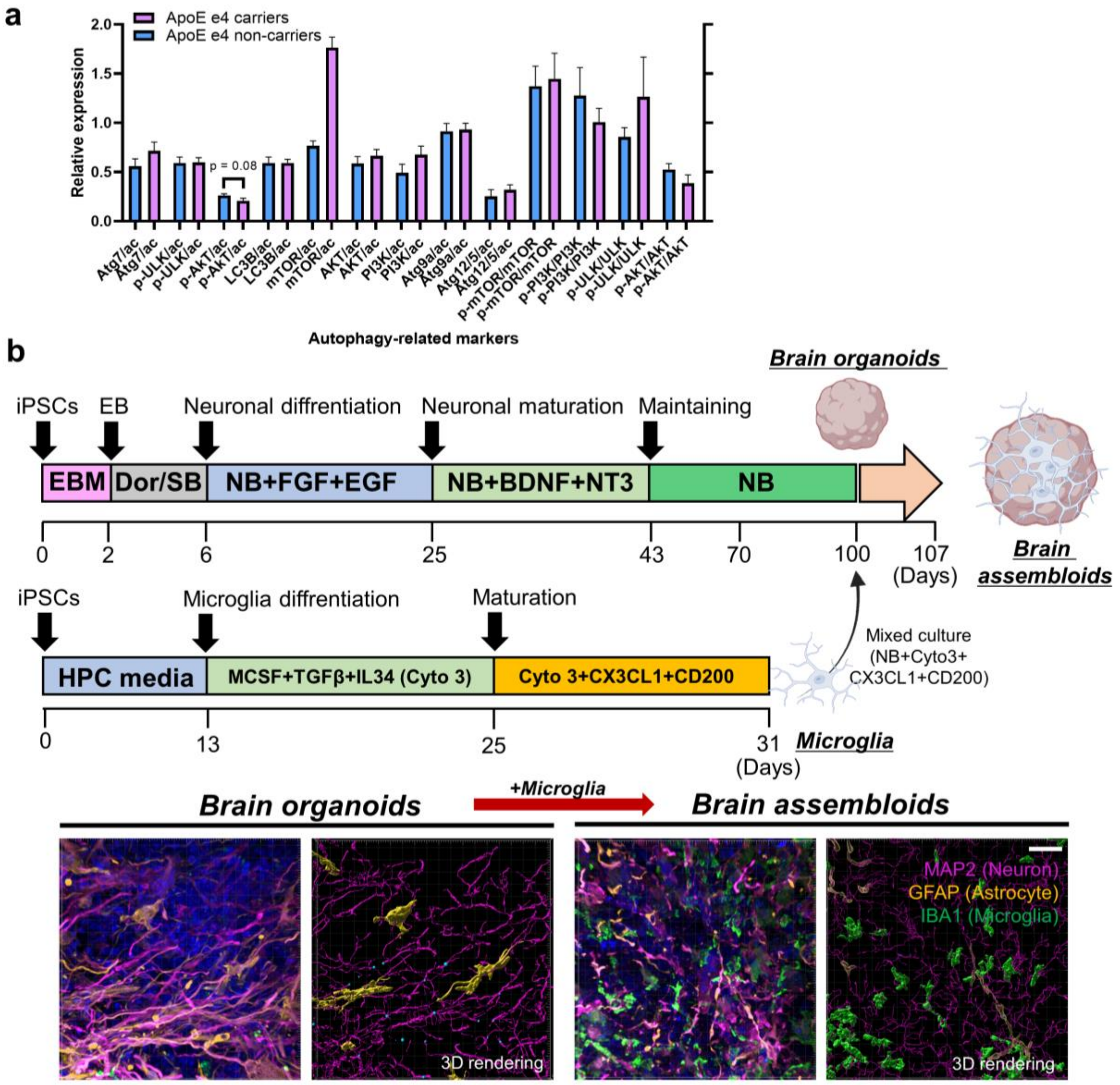

Figure S10. Comparison of the levels of autophagy-related markers in human PBMC samples between ApoE ε4 carriers vs ε4 non-carriers (only CN PiB-PET negative group included) and generation of brain organoids & assembloids. (a) No significances were observed (n = 15, ApoE ε4-positive CN with PiB-PET negative; n = 15, ApoE ε4-negative with PiB-PET negative). **(b)** Generation of brain organoids and assembloids. For more details, please see the method section. Images by spinning disk confocal microscope were processed with IMARIS software for 3D rendering. Scale bar = 20 μm,

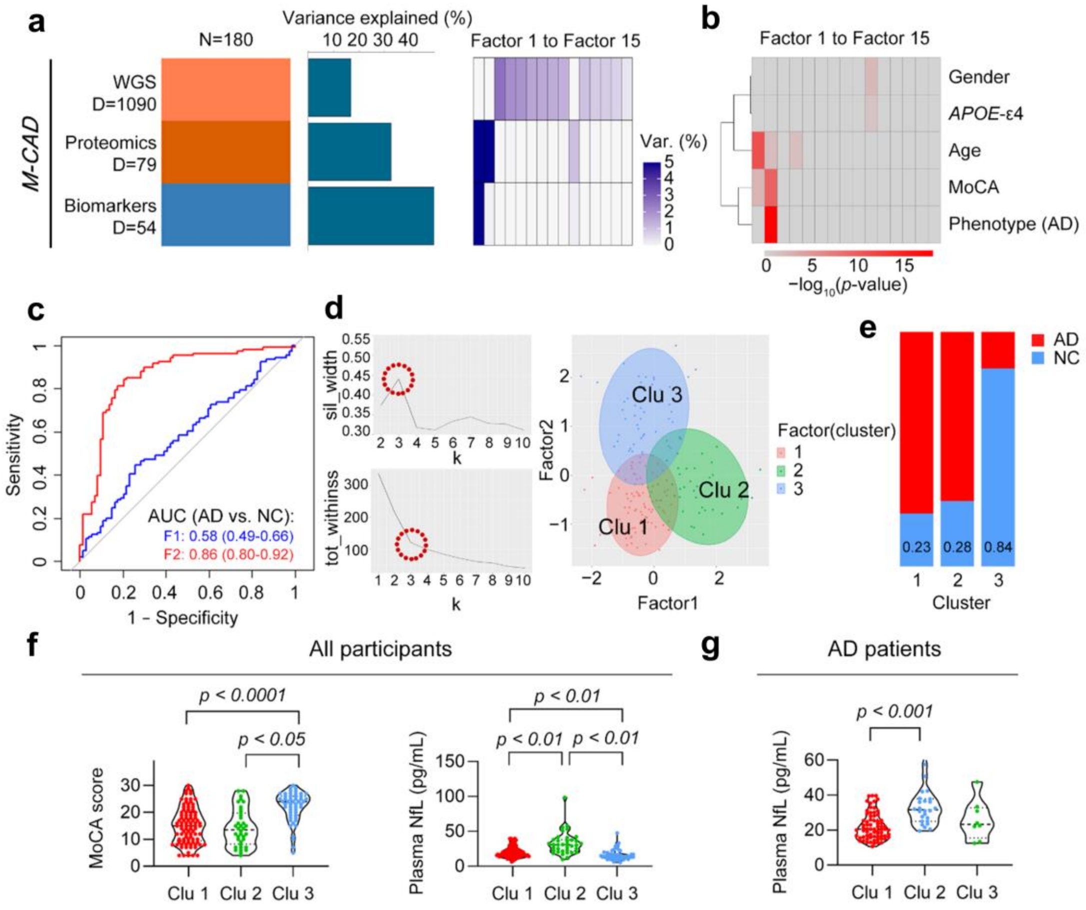

Figure S11. Multi-omics analysis of a Chinese Alzheimer's disease cohort. (a) Summary of the data evaluated by multi-omics analysis using the MOFA+ tool. The study included 180 total participants (*n* = 106 patients with AD, *n* = 74 CNs). The panel displays the types of dataset layers (left), the cumulative proportion of total variance explained (middle), and the proportion of variance explained by individual factors (right). **(b)** Heatmap of the association between individual factors and key cohort characteristics. Colormap indicates the significance of the correlation as measured by the *p*-value (in log10 scale). **(c)** ROC curve classifying AD based on factors associated with cognitive performance (Factor 1 and 2 denoted by the colors blue and red, respectively). AUC values (95% CI) are shown. **(d)** Silhouette and elbow plot analyses determined the number of clusters to use in the k-medoids clustering analysis of study participants (k = 3) using Factors 1 and 2. **(e)** Proportions of CNs and patients with AD in each cluster obtained from the k-medoids clustering analysis. Fractions of CNs are marked in the plot. **(f)** Comparison of MoCA scores (left) and plasma NfL levels (right) among all participants from different clusters. **(g)** Comparison of plasma NfL levels (right) among patients with AD from different clusters. For (F–G), robust regression test was adjusted for age, gender, and the top five principal components (also education level when testing MoCA). **Abbreviations:** AD, Alzheimer's disease; AUC, area under the ROC curve; CI, confidence interval; Clu, cluster; CNs, normal controls; M-CAD, Chinese AD cohort; MoCA, Montreal Cognitive Assessment; NfL, neurofilament light polypeptide; WGS, whole-genome sequencing.

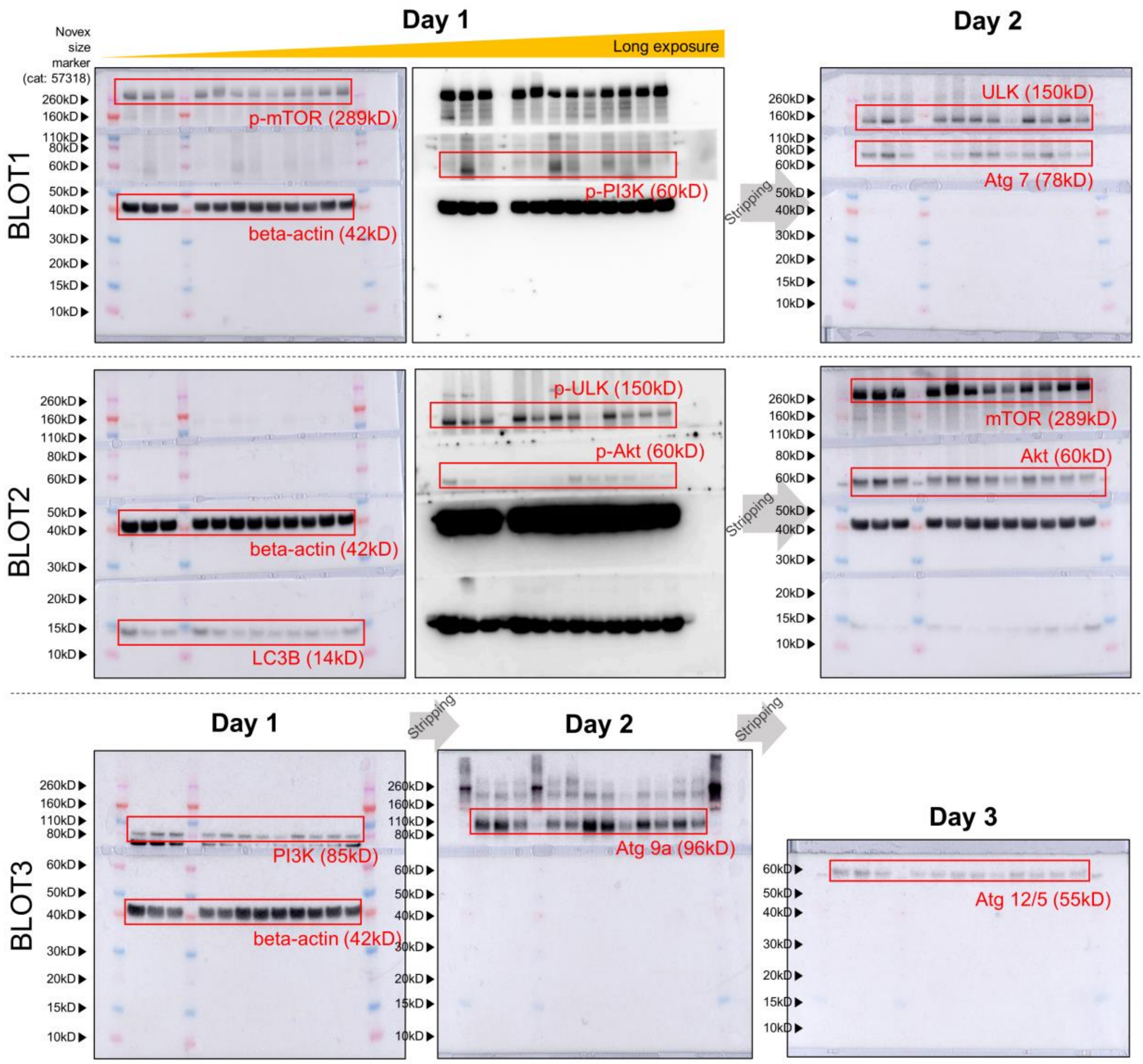

Figure S12. Full blots with a size marker (Novex size marker from Thermo scientific, cat: 57318) for autophagy-related molecules in PBMC samples (related to Figure 7). The same set of samples (n = 12, one cohort) was loaded into three blots (BLOT1 to BLOT3). For Figure 7, representative bands for beta-actin were extracted from BLOT1 (because samples in BLOT1, 2, and 3 were all equal), but quantification of each protein was performed using beta-actin bands from each blot (e.g., in BLOT3, the intensities of PI3K were normalized by the intensities of beta-actin in BLOT3). In total, 120 samples from 10 cohorts were quantified for the validation of autophagy-related molecules in PBMC samples.

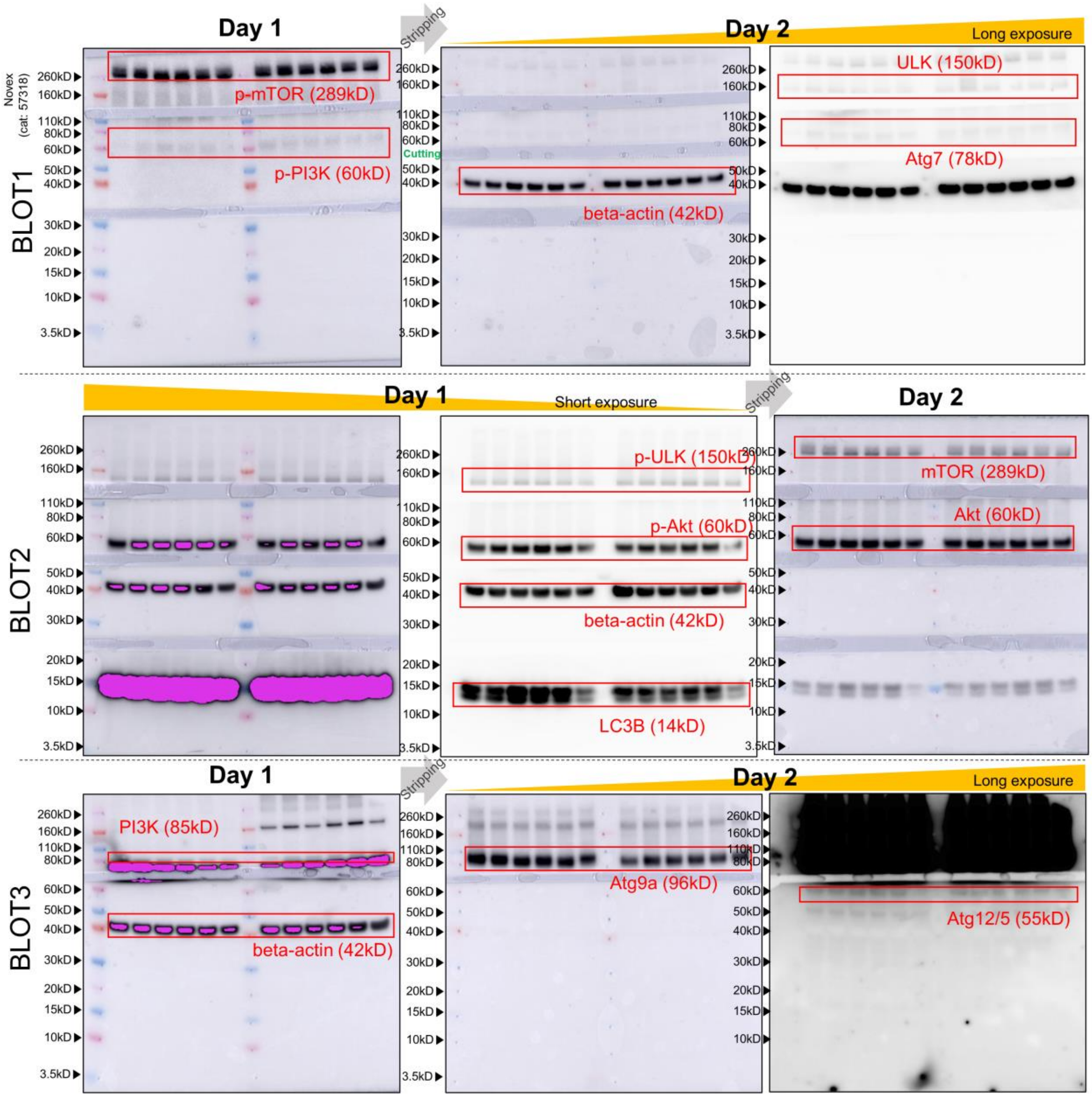

Figure S13. Full blots with a size marker (Novex size marker from Thermo scientific, cat: 57318) for autophagy-related molecules in CRISPR-Cas9-based ApoE isotype (ε3/ε3, parental line; ε4/ε4, isogenic line) brain organoid samples (related to Figure 7). The same set of samples (n = 12, one cohort) was loaded into three blots (BLOT1 to BLOT3). For Figure 7, representative bands for beta-actin were extracted from BLOT3 (because samples in BLOT1, 2, and 3 were all equal), but quantification of each protein was performed using beta-actin bands from each blot (e.g., in BLOT3, the intensities of PI3K were normalized by the intensities of beta-actin in BLOT3).

Supporting tables

Table S1. Demographic data of the participants of the study (n = 170; PiB+ participants)

Characteristics (n)	CN+ (40)	MCI+ (65)	DEM+ (65)	P-value
**Sex, M/F, n**	23/17	22/43	23/42	< 0.05†
**Age in years, mean ± SEM**	74.0 ± 1.0	73.2 ± 0.8	72.0 ± 1.0	0.377*
**Education, mean ± SEM**	12.4 ± 0.7	10.3 ± 0.5	10.1 ± 0.6	< 0.05*
**MMSE, mean ± SEM**	27.1 ± 0.3	21.5 ± 0.4	16.8 ± 0.5	< 0.001*
**MMSE z, mean ± SEM**	0.37 ± 0.1	−1.6 ± 0.2	−3.2 ± 0.2	< 0.001*
**CDR (n)**	0 (40)	0.5 (65)	0.5 (22), 1 (43)	< 0.001†
**ApoE4 status, $\varepsilon4^{+}/\varepsilon4^{-}$, n**	14/26	38/27	47/18	< 0.001†
**PiB (SUVR), mean ± SEM**	1.7 ± 0.05	1.9 ± 0.03	2.1 ± 0.04	< 0.001*

Abbreviations: ApoE, Apolipoprotein E; CDR, clinical dementia rating; CN, cognitively normal; DEM, clinically Alzheimer's disease dementia; MCI, mild cognitive impairment; MMSE, mini-mental state examination; n, number of participants; MMSE z score, a revised value of the MMSE score with consideration for age, gender, and education level; PiB, Pittsburgh compound B; SEM, standard error of the mean; SUVR, standardized uptake value ratio.

* Significance by one-way analysis of variance test (ANOVA).

† Significance by chi-squared test.

Table S2. Demographic characteristics of the Chinese Alzheimer's disease cohort (related to Figure S11)

**Chinese AD cohort**	**CN**	**AD**
n	74	106
Age, years (SD)	73.99 (4.59)	79.08 (6.25)
Sex, female (%)	48 (64.86%)	92 (86.79%)
Edu, years (SD)	9.09 (5.10)	4.36 (4.65)
MoCA score (SD)	23.85 (3.43)	12.22 (4.80)
APOE-ε4 allele frequency	14.86%	26.89%

Abbreviations: AD, Alzheimer's disease; CN, cognitively normal; Edu, education level; MoCA, Montreal Cognitive Assessment; SD, standard deviation.

Table S3. Demographic characteristics of the PBMC cohort (n = 120) (related to Figure 7)

Characteristics (n)	CN− (30)	CN+ (30)	MCI+ (30)	DEM+ (30)	P-value
**Sex, M/F, n**	13/17	16/14	10/20	10/20	0.332†
**Age in years, mean ± SEM**	67.8 ± 1.3	74.3 ± 1.2	74.3 ± 1.1	71.5 ± 1.6	< 0.001*
**Education, mean ± SEM**	11.3 ± 0.8	12.4 ± 0.8	10.5 ± 0.9	10.1 ± 0.9	0.249*
**MMSE, mean ± SEM**	26.9 ± 0.5	27.1 ± 0.3	21.1 ± 0.6	16.2 ± 0.5	< 0.001*
**MMSE z, mean ± SEM**	0.22 ± 0.2	0.39 ± 0.1	−1.8 ± 0.3	−3.5 ± 0.2	< 0.001*
**CDR (n)**	0 (30)	0 (30)	0.5 (30)	0.5 (9), 1 (21)	< 0.001†
**ApoE4 status, $\varepsilon4^{+}/\varepsilon4^{-}$, n**	15/15	12/18	15/15	15/15	0.825†
**PiB (SUVR), mean ± SEM**	1.1 ± 0.02	1.7 ± 0.07	1.9 ± 0.06	2.0 ± 0.05	< 0.001*

Abbreviations: ApoE, Apolipoprotein E; CDR, clinical dementia rating; CN, cognitively normal; DEM, clinically diagnosed Alzheimer's disease dementia; MCI, mild cognitive impairment; MMSE, mini-mental state examination; n, number of participants; MMSE z score, a revised value of the MMSE score considering age, sex, and education level; PiB, Pittsburgh compound B; SEM, standard error of the mean; SUVR, standardized uptake value ratio; − or +, PiB-PET positivity.

* Significance by one-way analysis of variance test (ANOVA).

† Significance by chi-squared test.

Table S4. Demographic characteristics of the iPSC-derived organoid cohort (n = 10) (related to Figure 7)

Characteristics (n)	CN− (5)	MCI+ (2)	DEM+ (3)	P-value
**Sex, M/F, n**	3/2	0/2	0/3	0.117†
**Age in years, mean ± SEM**	69.0 ± 3.9	75.5 ± 0.5	73.0 ± 1.7	0.507*
**Education, mean ± SEM**	8.0 ± 2.6	12.0 ± 0.0	7.3 ± 2.4	0.567*
**MMSE, mean ± SEM**	26.2 ± 0.6	20.5 ± 0.5	16.7 ± 0.9	< 0.001*
**MMSE z, mean ± SEM**	0.38 ± 0.5	−2.0 ± 0.2	−3.0 ± 0.3	< 0.001*
**CDR (n)**	0 (5)	0.5 (2)	0.5 (1), 1 (2)	0.182†
**ApoE4 status, $\varepsilon4^+/\varepsilon4^-$, n**	0/5	0/2	2/1	0.054†
**PiB (SUVR), mean ± SEM**	1.1 ± 0.04	2.1 ± 0.15	2.0 ± 0.15	< 0.001*

Abbreviations: ApoE, Apolipoprotein E; CDR, clinical dementia rating; CN, cognitively normal; DEM, clinically diagnosed Alzheimer's disease dementia; MCI, mild cognitive impairment; MMSE, mini-mental state examination; n, number of participants; MMSE z score, a revised value of the MMSE score considering age, sex, and education level; PiB, Pittsburgh compound B; SEM, standard error of the mean; SUVR, standardized uptake value ratio; − or +, PiB-PET positivity.

* Significance by one-way analysis of variance test (ANOVA).

† Significance by chi-squared test.

Table S5. Demographic data of the M-TPAD clusters

Characteristics (n)	Cluster 1 (56)	Cluster 2 (87)	Cluster 3 (27)	P-value
**Gender, M/F, n**	16/40	39/48	13/14	0.098†
**Age, years, mean ± SEM**	72.6 ± 1.0	73.3 ± 0.8	72.7 ± 1.2	0.848*
**Education, mean ± SEM**	10.6 ± 0.6	10.4 ± 0.5	11.9 ± 1.0	0.346*
**MMSE, mean ± SEM**	21.1 ± 0.8	20.6 ± 0.5	22.3 ± 0.9	0.343*
**MMSE z, mean ± SEM**	−1.7 ± 0.3	−1.9 ± 0.2	−1.5 ± 0.3	0.604*
**CDR, 0/0.5/1, n**	15/26/15	18/45/24	7/16/4	0.624†
**ApoE4 status, $\varepsilon4^{+}/\varepsilon4^{-}$, n**	36/20	48/39	15/12	0.533†
**PiB (SUVR), mean ± SEM**	1.9 ± 0.06	1.9 ± 0.04	2.0 ± 0.08	0.338*

Abbreviations: ApoE, apolipoprotein E; CDR, clinical dementia rating; MMSE, Mini-Mental State Examination; MMSE z score, a revised value of the MMSE score with consideration for age, gender, and education level; n, number of participants; PiB, Pittsburgh compound B; SEM, standard error of the mean; SUVR, standardized uptake value ratio.

* significance by one-way analysis of variance test (ANOVA).

† significance by chi-squared test.

Table S6. Demographic data of the M-IPAD clusters

Characteristics (n)	Cluster 1 (128)	Cluster 2 (33)	Cluster 3 (9)	P-value
**Gender, M/F, n**	49/79	16/17	3/6	0.518†
**Age, years, mean ± SEM**	72.7 ± 0.7	73.0 ± 1.2	76.7 ± 1.6	0.275*
**Education, mean ± SEM**	11.2 ± 0.4	9.3 ± 0.9	9.1 ± 2.2	0.087*
**MMSE, mean ± SEM**	21.2 ± 0.4	21.2 ± 1.0	18.2 ± 1.9	0.246*
**MMSE z, mean ± SEM**	−1.8 ± 0.2	−1.5 ± 0.4	−2.5 ± 0.5	0.312*
**CDR, 0/0.5/1, n**	29/71/28	10/12/11	1/4/4	0.201†
**ApoE4 status, $\varepsilon 4^{+}/\varepsilon 4^{-}$, n**	72/56	19/14	8/1	0.158†
**PiB (SUVR), mean ± SEM**	1.9 ± 0.03	1.9 ± 0.06	2.3 ± 0.17	0.009*

Abbreviations: ApoE, apolipoprotein E; CDR, clinical dementia rating; MMSE, mini-mental state examination; MMSE z score, a revised value of the MMSE score with consideration for age, gender, and education level; n, number of participants; PiB, Pittsburgh compound B; SEM, standard error of the mean; SUVR, standardized uptake value ratio.

* significance by one-way analysis of variance test (ANOVA).

† significance by chi-squared test.

Table S7. Details of ROC curve analyses (related to Figure 2)

	Characteristics (n)	M-TPAD		M-IPAD	
		Factor 1	Factor 2	Factor 1	Factor 2
**Cluster 1 vs. 2**	**AUC (95% CI)**	0.996 (0.966, 1.000)	0.620 (0.535, 0.700)	1.000 (0.977, 1.000)	0.795 (0.724, 0.854)
	**Significance (P)**	< 0.0001	0.0099	< 0.0001	< 0.0001
	**Sensitivity (%)**	93.10	51.72	100.00	63.64
	**Specificity (%)**	100.00	76.79	100.00	97.66
	**Youden index**	> −0.021	> 0.178	> 0.549	> 0.715
**Cluster 1 vs. 3**	**AUC (95% CI)**	0.845 (0.748, 0.915)	0.997 (0.951, 1.000)	0.657 (0.571, 0.736)	1.000 (0.973, 1.000)
	**Significance (P)**	< 0.0001	< 0.0001	0.0695	< 0.0001
	**Sensitivity (%)**	70.37	100.00	100.00	100.00
	**Specificity (%)**	91.07	98.21	30.47	100.00
	**Youden index**	> −0.143	> −0.316	> −0.589	> −1.805
**Cluster 2 vs. 3**	**AUC (95% CI)**	0.819 (0.736, 0.885)	0.998 (0.965, 1.000)	0.886 (0.749, 0.963)	1.000 (0.916, 1.000)
	**Significance (P)**	< 0.0001	< 0.0001	0.0001	< 0.0001
	**Sensitivity (%)**	66.67	100.00	77.78	100.00
	**Specificity (%)**	90.80	97.70	100.00	100.00
	**Youden index**	> −0.004	> −0.316	> −0.310	> −1.805

Abbreviations: AUC, area under the curve, CI, confidence interval; M-IPAD, multi-omics-based immune profiling model for Alzheimer’s disease; M-TPAD; multi-omics-based total profiling model for Alzheimer’s disease; ROC, receiver operating characteristic.

Table S8. Details of ROC curve analyses (related to Figure S6)

	Characteristics (n)	M-TPAD		M-IPAD	
		Factor 1	Factor 2	Factor 1	Factor 2
**Cluster 1 vs. 2**	**AUC (95% CI)**	0.992 (0.959, 1.000)	0.958 (0.909, 0.985)	0.857 (0.791, 0.909)	0.962 (0.918, 0.986)
	**Significance (P)**	< 0.0001	< 0.0001	< 0.0001	< 0.0001
	**Sensitivity (%)**	96.43	96.43	72.73	87.88
	**Specificity (%)**	98.04	86.27	94.12	92.44
	**Youden index**	> 0.504	> 0.446	> 0.375	> 0.289
**Cluster 1 vs. 3**	**AUC (95% CI)**	0.920 (0.835, 0.970)	0.988 (0.931, 1.000)	0.894 (0.828, 0.942)	0.780 (0.698, 0.848)
	**Significance (P)**	< 0.0001	< 0.0001	< 0.0001	< 0.0001
	**Sensitivity (%)**	88.46	96.15	100.00	100.00
	**Specificity (%)**	86.27	96.08	85.71	52.94
	**Youden index**	> 0.318	> 0.139	> 0.066	> 0.041
**Cluster 2 vs. 3**	**AUC (95% CI)**	0.814 (0.729, 0.882)	0.996 (0.960, 1.000)	0.906 (0.775, 0.974)	NA
	**Significance (P)**	< 0.0001	< 0.0001	< 0.0001	
	**Sensitivity (%)**	84.62	100.00	77.78	
	**Specificity (%)**	70.24	95.24	100.00	
	**Youden index**	> 0.213	> 0.199	> 0.493	

Abbreviations: AUC, area under the curve, CI, confidence interval; M-IPAD, multi-omics-based immune profiling model for Alzheimer’s disease; M-TPAD; multi-omics-based total profiling model for Alzheimer’s disease; NA, not applicable; ROC, receiver operating characteristic.

Table S9. List of interactions, pathways, and references related to Figure 6f

Labeling	Interactions (related pathways)	P/N regulation	References
a)	AGE-RAGE-glucose metabolism	N	Miranda ER et al., Nutrients. 2019 Feb 13;11(2):386. doi: 10.3390/nu11020386. PMID: 30781793; Riviere S et al., Glycoconj J. 1998 Oct;15(10):1039-42. doi: 10.1023/a:1006902428776. PMID: 10211709 Atsushi Araki et al., Geriatrics and Gerontology. 2004 Sep 9; doi: 10.1111/j.1447-0594.2004.00169.x
b)	KIT-PI3K-Akt	P	Vajravelu BN et al., PLoS One. 2015 Oct 16;10(10):e0140798. doi: 10.1371/journal.pone.0140798. PMID: 26474484
c)	AGE-RAGE-PI3K-Akt	N	Hou X et al., Cardiovasc Diabetol. 2014 Apr 14;13:78. doi: 10.1186/1475-2840-13-78. PMID: 24725502
d)	PI3K-Akt-mTOR	P	Heras-Sandoval D et al., Cell Signal. 2014 Dec;26(12):2694-701. doi: 10.1016/j.cellsig.2014.08.019. Epub 2014 Aug 28. PMID: 25173700
e)	PI3K-Akt-autophagy	N	Chen J et al., J Cell Biochem. 2013 Feb;114(2):245-9. doi: 10.1002/jcb.24362. PMID: 22930581
f)	mTOR-autophagy	N	Heras-Sandoval D et al., Cell Signal. 2014 Dec;26(12):2694-701. doi: 10.1016/j.cellsig.2014.08.019. Epub 2014 Aug 28. PMID: 25173700
g)	Thyroid-autophagy	P	Sinha RA et al., Cell Biosci. 2016 Jul 19;6:46. doi: 10.1186/s13578-016-0113-7. PMID: 27437098
h)	Hippocampal volume-glucose metabolism	N	Bateman RJ et al., N Engl J Med. 2012 Aug 30;367(9):795-804. doi: 10.1056/NEJMoa1202753. Epub 2012 Jul 11. Erratum in: N Engl J Med. 2012 Aug 23;367(8):780. PMID: 22784036
i)	Autophagy-ubiquitination	N	Kocaturk NM et al., Front Cell Dev Biol. 2018 Oct 2;6:128. doi: 10.3389/fcell.2018.00128. PMID: 30333975
j)	Autophagy-hippocampal volume	P	Glatigny M et al., Curr Biol. 2019 Feb 4;29(3):435-448.e8. doi: 10.1016/j.cub.2018.12.021. Epub 2019 Jan 17. PMID: 30661803
k)	BECN1-autophagy	P	Tong X et al., Differentiation. 2021 Aug 23;121:35-43. doi: 10.1016/j.diff.2021.08.003. Epub ahead of print. PMID: 34454349
l)	Autophagy-lipid metabolism	P	Singh R et al., Nature. 2009 Apr 30;458(7242):1131-5. doi: 10.1038/nature07976. Epub 2009 Apr 1. PMID: 19339967
m)	ITPR3-BECN1	N	Sica V et al., Mol Cell. 2015 Aug 20;59(4):522-39. doi: 10.1016/j.molcel.2015.07.021. PMID: 26295960
n)	COX-1-hippocampal volume	P	Tronel C et al., Int J Mol Sci. 2017 Apr 11;18(4):802. doi: 10.3390/ijms18040802. PMID: 28398245; Bateman RJ et al., N Engl J Med. 2012 Aug 30;367(9):795-804. doi: 10.1056/NEJMoa1202753. Epub 2012 Jul 11. Erratum in: N Engl J Med. 2012 Aug 23;367(8):780. PMID: 22784036
o)	ACE-lipid metabolism	P	De Cesaris R et al., Cardiology. 1993;83(3):165-72. doi: 10.1159/000175965. PMID: 8281530
p)	ACE-blood controls	P	Henriksen EJ et al., J Cell Physiol. 2003 Jul;196(1):171-9. doi: 10.1002/jcp.10294. PMID: 12767053

Supporting methods

Single nucleotide variants (SNVs) dataset by targeted sequencing

For the SNVs dataset, high-quality SNVs on AD susceptibility genes from targeted sequencing data, which was previously published by the Korean Brain Aging Study for Early Diagnosis and Prediction of Alzheimer's disease (KBASE) research group, were used [1]. Briefly, 132 genes were selected as per the following criteria: (1) genes from previous genome-wide association studies for AD, (2) genes from the Online Mendelian Inheritance in Man database (https://www.ncbi.nlm.nih.gov/omim), (3) genes from the Kyoto Encyclopedia of Genes and Genomes (KEGG) pathway database for AD (www.genome.jp/kegg/pathway.html), (4) manually selected genes related to AD. The whole gene list, sequencing, and variant calling information can be found in the previous study [1]. For this multi-omics study, we selected dpSNP reference (rs)-number assigned 4,140 SNVs and then sorted SNVs found in not less than 5% but not more than 95% of the participants (125 genes, 1,133 SNVs). For the multi-omics based-immune profiling model for AD (M-IPAD), we singled out specific immune-related genes (76 genes) by Immport DB (www.immport.org) again and used their SNVs (658 SNVs) as inputs for the analyses (**Figure 1** and see **Figure S1-S2**) [2].

miRNA transcriptome dataset

For the miRNA dataset, Nanostring nCounter miRNA analysis (NanoString Technologies, Seattle, WA, USA) was performed by ChunLab (Seoul, South Korea) according to the manufacturer's protocol [3]. Briefly, the workflow for the nCounter miRNA assay consists of two steps (day 1, manual processing, miRNA sample preparation and hybridization; day 2, automated post-hybridization processing using the nCounter Prep Station and Digital

analyser). At day 1, purified total RNA from human blood samples was used for the miRNA-specific preparation. Using nCounter miRNA Sample Prep Kit, 3 μL of total RNA was mixed with 3.5 μL of annealing master mix (13 μL of Annealing buffer + 26 μL of nCounter miRNA Tag Reagent + 6.5 μL of 1:500 miRNA Assay Controls). After spinning down, the strip was placed in a thermal cycler and the annealing protocol was initiated. Next, the ligand protocol was initiated with the ligand master mix (19.5 μL of PEG solution + 13 μL of ligand buffer). Immediately after the ligation protocol, 1 μL of Ligation Clean-Up Enzyme was added to each reaction. After returning the tubes to the thermal cycler, the purification protocol was initiated, and after that, 40 μL of RNAse-free water was added to each sample. After completion of the purification step, hybridization of miRNA CodeSet was performed. First, 20 μL of master mix (130 μL of miRNA Reporter CodeSet + 130 μL of hybridization buffer) was added to each of the tubes and 5 μL of prepared samples from the miRNA sample preparation step were mixed. After adding 5 μL of Capture Probeset, each tube was immediately moved to 65 °C. The samples were incubated for hybridization for at least 12 h. At day 2, the samples were removed from the thermal cycler, and the post-hybridization process with nCounter Prep Station (cat: NCT-PREP-120) and digitalized analysis (Digital Analyzer; cat: NCT-DIGT-120) were performed immediately. For this multi-omics study, all values were normalized with the endogenous control (GAPDH), tested for multicollinearity (variance inflation factor < 10), and followed standardized quality-control steps as described in **Figure S2**.

Proteomics dataset

The proteomics dataset was obtained from our previous report which revealed proteomics-based biomarkers for AD using the tandem mass tag (TMT)-quantitative approaches. All methods (immune-affinity depletion, TMT-labelling, pH reverse-phase liquid

chromatography fractionation, N-glycopeptide enrichment, and liquid chromatography with tandem mass spectrometry analysis) were previously described [4]. The relative abundance of the peptides was quantified by the normalization with a universal control, and 1.3-fold changed values (compared with median value of universal 1 and 2) were considered as differentially expressed (DE) peptides. Proteins at least having two DE peptides were identified as the differentially expressed protein (DEPs). For multi-omics analysis, all values were tested for multicollinearity (variance inflation factor < 10) and followed standardized quality-control steps as described in **Figure S2**. For the M-IPAD, we further sorted out specific immune-related targets (183 proteins) from the whole targets (398 proteins) using Immport DB (www.immport.org) and used them as inputs for the analyses (**Figure 1** and see **Figure S1-S2**) [2].

Blood-based biomarkers dataset

The blood-based biomarker dataset (total 136 targets) included immunoassay-based proteins (60 targets from cytokine array, 16 targets from western blotting, 11 targets from xMAP or Simoa assay, two targets from *QPLEX* Alz-plus assay, five targets from enzyme-linked immunosorbent assay, and 16 targets from flow cytometry) and other 26 blood test factors that are measured in medical health check-ups (colorimetric, immunoturbidimetric assay, inductively coupled plasma mass spectrometry, high-performance liquid chromatography, and chemiluminescence immunoassay). See the target list and more details in **Supplementary data**. For immunoassay-based proteins, cytokine profile in the human serum samples was measured using Proteome Profiler Human XL Cytokine Array Kit according to the manufacturer's instruction (ARY022B; R&D systems, Minneapolis, MN, USA). For specific autophagy-related cytokines in peripheral blood mononuclear cells, additional western blotting was performed using cell lysates of these cells with the same protocol as in our

previous study [5]. The levels of plasma Aβ 42, 40, 42/40 ratio were quantified by INNO-BIA plasma Aβ forms assay (81578; Fujirebio, Ghent, Belgium) using xMAP technology (bioplex 200; Bio-Rad, Hercules, CA, USA) according to the manufacturer's guidelines [6]. Plasma total tau (Simoa Tau 2.0 kit, 101552; Quanterix, Billerica, MA, USA) and phosphorylated tau (Simoa p-tau Thr 181 kit, 103377; Quanterix) were measured by Simoa HD-1 analyser (Quanterix) as per the manufacturer's instruction [7]. Six combinative biomarkers (ratio-based) between the plasma Aβ and t-tau or p-tau were added since they have also been reported as biomarkers for AD [6-8]. Two targets (galectin-3 binding protein, LGALS3BP; periostin, POSTN) were quantified by *QPLEX* Alz-plus assay (Quantamatrix, Seoul, South Korea) as per the manufacturer's instruction [9]. Five targets (VE-cadherin, thrombospondin 1, angiotensin converting enzyme 1, galectin-3, and LGALS3BP/gal-3 ratio) were measured by enzyme-linked immunosorbent assays according to each manufacturer's guidelines (VE-cadherin, DCADV0; thrombospondin 1, DTSP10; angiotensin converting enzyme 1, DACE00; galectin-3, DGAL30; all kits from R&D systems. Seventeen targets were the population of immune-cells (e.g., T, B, and natural killer cells) and measured by flow-cytometry with the same protocol as in our previous study [10]. The blood test factors, which consisted of general factors from blood test in routine health check-ups, were measured by various methods and performed at the Seoul Clinical Laboratories (SCL, Gyeonggido, South Korea) as we previously reported [11], except for the activity of acetylcholinesterase (ab235937; Abcam, Cambridge, UK). For this multi-omics study, all values were tested for multicollinearity (variance inflation factor < 10) and followed standardized quality-control steps as described in **Figure S2**.

Statistical analysis for the downstream analyses beyond MOFA+

For clustering, we set and applied standardized criteria to the trained multi-omics factors

(**Figure 2**). The elbow plot and average silhouette analysis were performed using *ggplot2* R package (version 3.3.3), to determine the optimal number of clusters and factor-combination [12]. Next, we classified our observations into appropriate clusters by k-medoids algorithm (partitioning around medoids; PAM algorithm), a robust alternative to k-means clustering [13]. MedCalc Software ver. 20.009 (Ostend, Belgium) and Prism ver. 8.0 (GraphPad Software, San Diego, CA, USA) were used for further downstream analyses. Receiver operating characteristic (ROC) curve analysis followed by logistic regression analysis was performed with or without the correction of covariates. Numerical data were tested using ANOVA with Tukey's post-hoc test or independent *t*-test. Categorical data, such as ApoE allele or sex, were tested by Chi-square test. Pearson's correlation analysis was conducted for identifying the association between two continuous variables.

STRING and context-specific protein-protein interaction network analysis

To identify functional interaction between our top-rated targets, STIRNG PPI network analysis was performed (https://string-db.org) (**Figure S7**) [14]. We inputted the top-rated targets (from **Figure 3a or 4a**; all SNPs from the targeted sequencing data were converted into their gene names; miRNAs were excluded because they could not be converted into gene names) into the STRING PPI software. Then the number of nodes, edges, average node degree, and PPI enrichment *p*-values were calculated and used to validate the association between our top-rated targets. Certain predicted functional partners were automatically added to each network model. Context-specific PPI analysis was performed using Integrated Interactions Database (IID; http://iid.ophid.utoronto.ca/) (interaction conditions: cancer and developmental stages were excluded, whereas disease of anatomical entry, mental health, and metabolism were included; PPIs were only assigned to a disease when both genes were expressed) including targets of miRNAs (**Figure S8**). In addition, all targets of top-rated

miRNAs were converted into gene names from the public miRDB database (http://mirdb.org) in the context-specific PPI model. All information on PPI and annotations from each multi-omics factor (MOF) was uploaded to https://github.com/jcparkgithub/multiomics_for_mtpad_mipad.

Comparative MOFA+ analysis between cognitively normal (CN+) and AD patients within the Hong Kong cohort

To use MOFA+ as discrimination tool between CN+ vs AD, a Hong Kong cohort comprising 74 CN+ and 106 AD was used. This dataset included plasma proteins (Aβ, phosphorylated tau, and total tau, among others), blood proteomics, and whole-genome sequencing data. Only the proteins and genomic variants that overlapped with M-TPAD were used for the analysis. The detailed methods for DNA and plasma extraction from blood samples, plasma protein measurement, proteomics, and whole-genome sequencing were described elsewhere [15].

Enriched pathway analysis

Our targets that showed significant increase or decrease rate (threshold > 0.5 or < -0.5, respectively) compared with the average value of the whole cohort were used as input to reveal important pathways of each cluster. Six public databases were used (KEGG_2019_human, GO_molecular_function_2018, GO_cellular_component_2018, GO_biological_process_2018, BioCarta_2016, Reactome_2016). To use miRNA database as one of the inputs, targets of miRNAs that showed over 95 points (target score) were selected from miRDB (http://mirdb.org). The overlapping genes were counted and displayed as circle size, and the vacillating tendency of each pathway was illustrated in red (upward) and blue (downward). Pathways were selected using a false discovery rate (FDR)-adjusted p-value $<$ 0.05.

Multiple clustering analysis (MCA) and key-driver analysis (KDA)

To identify key drivers within our top-rated targets, MCA followed by KDA was performed with a network model from heterogenous network-based dataset for AD (HENA) and the analysis for multiscale clustering of geometrical network (MEGENA) [16]. We used network information from HENA, which is constructed by integrating multi-omics (i.e., PPI, gene expression, epistasis, GWAS). From the 31,826 nodes and 63,084,850 edges throughout the HENA network model, 14,674 nodes and 21,368,289 edges were selected using interaction scores (> 0.5 or < −0.5). First, for the network module clustering, MCA was performed according to the algorithm in the MEGENA database. Three multiple perspectives were considered as main aspects for identifying locally coherent clusters: i) optimized compactness of each cluster by the shortest path distances, ii) optimized local clustering structure using local path index, and iii) maintenance of optimal balances using overall modularity. A total of 504 modules were finally selected. Second, KDA was performed to identify key driver genes (hub genes) according to their enrichment scores and FDR-adjusted *p*-values ($p < 0.05$). A total of 314 clusters were selected among 504 modules, and 3,631 key driver nodes were finally singled out, considering selected modules and their co-expression network. The key driver nodes were used for the selection of the key drivers in our multi-omics models.

Immunohistochemistry and 3D rendering using IMARIS software

Brain organoids or assembloids were immersed in 4% PFA solution (4°C overnight), washed with PBS, and immersed again in 30% sucrose at 4°C for 72 hours. They were frozen in FSC 22 Compound solution (Leica, Wetzlar, Germany) and cryo-sectioned for immunohistochemistry. The sectioned slices were permeabilized with 0.3% Triton X-100 in PBS and blocked using 5% bovine serum albumin (BSA) in PBS for 1 hour at room-temperature. Next, primary antibodies were applied at 4°C overnight, and fluorophore-

conjugated secondary antibodies in 3% BSA solution were treated for 1 hour at RT. The tissues were mounted on glass-slides. Confocal images were acquired using Spinning disc confocal microscopy (CSU-X1, Yokogawa Electric, Tokyo, Japan). Image visualization, 3D rendering, filament tracking, and quantification of LC3B per single cell (neuron, astrocyte, microglia) were performed by IMARIS software (Bitplane, Zurich, Switzerland).

Supplementary references

[1] J. Seo, M. S. Byun, D. Yi, J. H. Lee, S. Y. Jeon, S. A. Shin, Y. K. Kim, K. M. Kang, C. H. Sohn, G. Jung, J. C. Park, S. H. Han, J. Byun, I. Mook-Jung, D. Y. Lee, M. Choi, K. R. Group, *Alzheimers Res Ther* **2020**, *12* (1), 156, https://doi.org/10.1186/s13195-020-00722-2.

[2] S. Bhattacharya, S. Andorf, L. Gomes, P. Dunn, H. Schaefer, J. Pontius, P. Berger, V. Desborough, T. Smith, J. Campbell, E. Thomson, R. Monteiro, P. Guimaraes, B. Walters, J. Wiser, A. J. Butte, *Immunol Res* **2014**, *58* (2-3), 234, https://doi.org/10.1007/s12026-014-8516-1.

[3] J. H. Kim, Y. H. Cho, Y. C. Hong, *Environ Int* **2020**, *141*, 105791, https://doi.org/10.1016/j.envint.2020.105791.

[4] J. C. Park, S. H. Han, H. Lee, H. Jeong, M. S. Byun, J. Bae, H. Kim, D. Y. Lee, D. Yi, S. A. Shin, Y. K. Kim, D. Hwang, S. W. Lee, I. Mook-Jung, *Prog Neurobiol* **2019**, *183*, 101690, https://doi.org/10.1016/j.pneurobio.2019.101690.

[5] J. C. Park, S. H. Baik, S. H. Han, H. J. Cho, H. Choi, H. J. Kim, H. Choi, W. Lee, D. K. Kim, I. Mook-Jung, *Aging Cell* **2017**, *16* (1), 149, https://doi.org/10.1111/acel.12530.

[6] J. C. Park, S. H. Han, H. J. Cho, M. S. Byun, D. Yi, Y. M. Choe, S. Kang, E. S. Jung, S. J. Won, E. H. Kim, Y. K. Kim, D. Y. Lee, I. Mook-Jung, *Alzheimers Res Ther* **2017**, *9* (1), 20, https://doi.org/10.1186/s13195-017-0248-8.

[7] J. C. Park, S. H. Han, D. Yi, M. S. Byun, J. H. Lee, S. Jang, K. Ko, S. Y. Jeon, Y. S. Lee, Y. K. Kim, D. Y. Lee, I. Mook-Jung, *Brain* **2019**, *142* (3), 771, https://doi.org/10.1093/brain/awy347.

[8] a) J. A. C. Sterne, J. Savovic, M. J. Page, R. G. Elbers, N. S. Blencowe, I. Boutron, C. J. Cates, H. Y. Cheng, M. S. Corbett, S. M. Eldridge, J. R. Emberson, M. A. Hernan, S. Hopewell, A. Hrobjartsson, D. R. Junqueira, P. Juni, J. J. Kirkham, T. Lasserson, T. Li, A. McAleenan, B. C. Reeves, S. Shepperd, I. Shrier, L. A. Stewart, K. Tilling, I. R. White, P. F. Whiting, J. P. T. Higgins, *BMJ* **2019**, *366*, l4898, https://doi.org/10.1136/bmj.l4898; b) C. Ritchie, N. Smailagic, A. H. Noel-Storr, O. Ukoumunne, E. C. Ladds, S. Martin, *Cochrane Database Syst Rev* **2017**, *3*, CD010803, https://doi.org/10.1002/14651858.CD010803.pub2.

[9] J. C. Park, K. S. Jung, J. Kim, J. S. Jang, S. Kwon, M. S. Byun, D. Yi, G. Byeon, G. Jung, Y. K. Kim, D. Y. Lee, S. H. Han, I. Mook-Jung, *Alzheimers Res Ther* **2021**, *13* (1), 12, https://doi.org/10.1186/s13195-020-00751-x.

[10] J. C. Park, *Research Square* **2021**, https://doi.org/10.21203/rs.3.rs-312269/v1.

[11] H. J. Kim, J. C. Park, K. S. Jung, J. Kim, J. S. Jang, S. Kwon, M. S. Byun, D. Yi, G. Byeon, G. Jung, Y. K. Kim, D. Y. Lee, S. H. Han, I. Mook-Jung, *Exp Mol Med* **2021**, https://doi.org/10.1038/s12276-021-00638-3.

[12] a) A. Abrol, E. Damaraju, R. L. Miller, J. M. Stephen, E. D. Claus, A. R. Mayer, V. D. Calhoun, *Neuroimage* **2017**, *163*, 160, https://doi.org/10.1016/j.neuroimage.2017.09.020; b) A. Lengyel, Z. Botta-Dukat, *Ecol Evol* **2019**, *9* (23), 13231, https://doi.org/10.1002/ece3.5774.

[13] S. Fayech, N. Essoussi, M. Limam, *BioData Min* **2009**, *2* (1), 3, https://doi.org/10.1186/1756-0381-2-3.

[14] D. Szklarczyk, A. L. Gable, D. Lyon, A. Junge, S. Wyder, J. Huerta-Cepas, M. Simonovic, N. T.

Doncheva, J. H. Morris, P. Bork, L. J. Jensen, C. V. Mering, *Nucleic Acids Res* **2019**, *47* (D1), D607, https://doi.org/10.1093/nar/gky1131.

[15] Y. Jiang, X. Zhou, F. C. Ip, P. Chan, Y. Chen, N. C. H. Lai, K. Cheung, R. M. N. Lo, E. P. S. Tong, B. W. Y. Wong, A. L. T. Chan, V. C. T. Mok, T. C. Y. Kwok, K. Y. Mok, J. Hardy, H. Zetterberg, A. K. Y. Fu, N. Y. Ip, *Alzheimers Dement* **2021**, https://doi.org/10.1002/alz.12369.

[16] a) E. Sugis, J. Dauvillier, A. Leontjeva, P. Adler, V. Hindie, T. Moncion, V. Collura, R. Daudin, Y. Loe-Mie, Y. Herault, J. C. Lambert, H. Hermjakob, T. Pupko, J. C. Rain, I. Xenarios, J. Vilo, M. Simonneau, H. Peterson, *Sci Data* **2019**, *6* (1), 151, https://doi.org/10.1038/s41597-019-0152-0; b) W. M. Song, B. Zhang, *PLoS Comput Biol* **2015**, *11* (11), e1004574, https://doi.org/10.1371/journal.pcbi.1004574; c) L. Shu, Y. Zhao, Z. Kurt, S. G. Byars, T. Tukiainen, J. Kettunen, L. D. Orozco, M. Pellegrini, A. J. Lusis, S. Ripatti, B. Zhang, M. Inouye, V. P. Makinen, X. Yang, *BMC Genomics* **2016**, *17* (1), 874, https://doi.org/10.1186/s12864-016-3198-9.
